# Adults from Kisumu, Kenya have robust γδ T cell responses to *Schistosoma mansoni*, which are modulated by tuberculosis

**DOI:** 10.1371/journal.pntd.0008764

**Published:** 2020-10-12

**Authors:** Taryn A. McLaughlin, Jeremiah Khayumbi, Joshua Ongalo, Daniel Matete, Joan Tonui, Benson Muchiri, Loren E. Sasser, Angela Campbell, Salim Allana, Samuel Gurrion Ouma, Felix Odhiambo Hayara, Neel R. Gandhi, Cheryl L. Day

**Affiliations:** 1 Emory Vaccine Center, Emory University, Atlanta, Georgia, United States of America; 2 Center for Global Health Research, Kenya Medical Research Institute, Kisumu, Kenya; 3 Department of Epidemiology, Rollins School of Public Health, Emory University, Atlanta, Georgia, United States of America; 4 Division of Infectious Diseases, Department of Medicine, Emory University School of Medicine, Atlanta, Georgia, United States of America; 5 Department of Microbiology & Immunology, Emory University School of Medicine, Atlanta, Georgia, United States of America; University of Manchester, UNITED KINGDOM

## Abstract

*Schistosoma mansoni* (SM) is a parasitic helminth that infects over 200 million people and causes severe morbidity. It undergoes a multi-stage life cycle in human hosts and as such stimulates a stage-specific immune response. The human T cell response to SM is complex and varies throughout the life cycle of SM. Relative to the wealth of information regarding the immune response to SM eggs, little is known about the immune response to the adult worm. In addition, while a great deal of research has uncovered mechanisms by which co-infection with helminths modulates immunity to other pathogens, there is a paucity of data on the effect of pathogens on immunity to helminths. As such, we sought to characterize the breadth of the T cell response to SM and determine whether co-infection with *Mycobacterium tuberculosis* (Mtb) modifies SM-specific T cell responses in a cohort of HIV-uninfected adults in Kisumu, Kenya. SM-infected individuals were categorized into three groups by Mtb infection status: active TB (TB), Interferon-γ Release Assay positive (IGRA+), and Interferon-γ Release Assay negative (IGRA-). U.S. adults that were seronegative for SM antibodies served as naïve controls. We utilized flow cytometry to characterize the T cell repertoire to SM egg and worm antigens. We found that T cells had significantly higher proliferation and cytokine production in response to worm antigen than to egg antigen. The T cell response to SM was dominated by γδ T cells that produced TNFα and IFNγ. Furthermore, we found that in individuals infected with Mtb, γδ T cells proliferated less in response to SM worm antigens and had higher IL-4 production compared to naïve controls. Together these data demonstrate that γδ T cells respond robustly to SM worm antigens and that Mtb infection modifies the γδ T cell response to SM.

## Author summary

Schistosomiasis, a disease caused by parasitic helminths including *Schistosoma mansoni* (SM), affects hundreds of millions of people globally. SM undergoes a complex life cycle within humans resulting in adult worm pairs that release eggs into the circulatory system. The human immune response to SM, especially to adult worms, is not well characterized. In addition, the impact of co-infections, which are common in SM endemic regions, on the immune response to SM is unknown. In this study, we first sought to characterize the T cell response to different stages of the SM life cycle. We next evaluated whether T cell responses to SM were altered in the setting of co-infection with *Mycobacterium tuberculosis*, the bacteria that causes tuberculosis. We determined that human T cell responses to SM adult worm antigen are more robust than to SM egg antigen. This response is dominated by a non-classical T cell subset of γδ T cells producing IFNγ and TNFα. Lastly, we found that the ability of γδ T cells to proliferate in response to SM worm was lower in individuals with tuberculosis compared to naïve controls. This study provides novel insights into the immune response to SM and how tuberculosis may impair SM immunity.

## Introduction

Though considered a neglected tropical disease (NTD), helminthiasis is a serious global health burden. Over 1 billion people worldwide are estimated to be infected with one or more helminth species [1]. The most common helminth infections are schistosomes, lymphatic filarial worms, and the soil transmitted helminths ascaris, trichuris, and hookworm. Each of these individually infects hundreds of millions of people globally [1]. While not normally fatal, infections with helminths cause a great deal of morbidity and are collectively responsible for 26 million Disability Adjusted Life Years (DALYs) [1].

People in Sub-Saharan Africa are disproportionately impacted by helminth infections [2]. This is particularly true for schistosomiasis, which affects over 200 million people globally, 90% of whom reside in Sub-Saharan Africa [2]. Indeed, it is estimated that one in four people in Sub-Saharan Africa is infected with a schistosome species. The mortality rate for *Schistosoma mansoni* (SM), which is only one of five schistosome species known to infect humans, is estimated at 130,000 per year in Sub-Saharan Africa alone [3].

As with most helminths, SM has a complex life cycle which involves both a human and intermediate host [4]. Humans become infected when they come into contact with fresh water containing the infectious larval schistosomes, known as cercariae, that penetrate the skin. Within the human host, SM larvae migrate through the body, passing through a variety of tissues including the lung, as they develop into adult worms. When adult worms reach sexual maturity, they take up residence in the portal vein. If left untreated, adult mating pairs can reside in the bloodstream for years releasing hundreds of eggs every day [4]. These eggs are passed through the endothelium into the gastrointestinal tract and are shed in stool. Eggs that come into contact with fresh water hatch and release miracidia, which are the infectious form for the snail intermediate host. Parasites replicate asexually in snails, releasing cercariae back into fresh water.

In contrast to other helminths, which are predominantly type 2, the immune response to SM is stage specific. In experimental models, the migrating larval stage of the worm elicits a type 1 response which gives rise to a type 2 response upon egg laying and transitions to a regulatory phenotype when the infection becomes chronic [5,6]. Much more is known about the immune response to SM eggs than either the larvae or adult worms. This is largely due to the role the eggs play in immunopathology and their ability to modulate antigen presentation and CD4 T cell phenotypes [7]. Most studies on human SM T cell immunity have focused on CD4 T cells [7]. Very little is therefore known about human CD8 T cell responses and even less about non-classical T cell responses, such as γδ T cells, which can have diverse roles during helminth infections.

γδ T cells in humans are divided based on their V chain expression and anatomical location, which in turn dictate their function. Intraepithelial lymphocytes (IELs) are largely Vδ1 cells and are involved in surveillance and maintenance of barrier tissues [8,9]. IELs are involved in the expulsion of the helminths *Nippostrongylus brasiliensis* and *Trichinella spiralis* from the intestines of infected mice [10–12]. Hepatic γδ T cells are often Vγ4 cells and secrete the cytokine IL-17 and/or IL-10 [13]. Studies in mice indicate a role for IL-17 producing γδ T cells in liver fibrosis and immune pathology during infection with *Schistosoma japonicum* [14–17] and SM [18]. They can either be pathogenic as in the case of *S*. *japonicum*, or protective in the case of *Listeria monocytogenes* [13]. In the blood, γδ T cells are predominantly Vγ2Vδ2 (alternatively called Vγ9Vδ2) [19–21]. They canonically proliferate and produce IFNγ in response to phosphoantigens produced by pathogens [22,23]. Vγ2Vδ2 T cell populations expand in the peripheral blood of people in response to a variety of infections [22,24] including SM [25]. While it is clear that γδ T cells respond to infection with helminths, whether they respond to helminth antigen directly or are merely bystander cells is still unknown.

Peripheral γδ T cells respond to phosphoantigens, which are produced by a variety of microbes [22,26]. For this reason, the role of γδ T cells during SM infection is of particular interest because SM is co-endemic with a variety of pathogens known to elicit γδ T cell responses including *Mycobacterium tuberculosis* (Mtb) [27,28]. Co-infection with SM and Mtb is common throughout the world, particularly in sub-Saharan Africa, and may impact the immune response to both pathogens. A great deal of research has been conducted evaluating the impact of helminth infections on immunity to Mtb [28,29]; however, the effect of Mtb on the immune response to helminths is less clear. There are no published studies that have evaluated the impact of Mtb infection on SM-specific T cell responses.

In this study, we characterized the T cell repertoire to two SM antigens from distinct stages of the SM life cycle. We enrolled individuals in groups defined by Mtb and SM infection status: SM-naïve controls (N) from the US, and SM-infected individuals from Kenya who were further stratified into active TB (TB), Interferon-γ Release Assay positive (IGRA+), and Interferon-γ Release Assay negative (IGRA-). In each group we examined the ability of T cells to produce cytokines and proliferate in response to adult worm antigens compared to egg antigens. In addition, we measured the impact of Mtb infection status on T cell functions. We hypothesized that Mtb infection would dampen the immune response to SM.

## Methods

### Study population

Participants ≥18 years old were recruited in either Kisumu County, Kenya, as described previously [30] or Atlanta, GA, United States. Individuals in Kenya were recruited from two community based health clinics located in Kisumu City and Kombewa. All participants from Kisumu were SM+ by Kato-Katz stool microscopy. They were enrolled based on Mtb infection status into three groups: active TB disease (TB), IGRA+, and IGRA- controls. Patients with drug-sensitive active pulmonary TB disease were symptomatic individuals with a positive GeneXpert MTB/RIF result and a positive culture for Mtb growth. Healthy asymptomatic individuals with no previous history of TB disease or treatment were evaluated by QuantiFERON-TB Gold In-Tube (QFT) assay: those with a positive QFT result (TB Ag-Nil >0.35 IU IFNγ/ml) were defined as IGRA+; those with a negative QFT result (TB Ag-Nil <0.35 IU IFNγ/ml) were defined as IGRA-. All IGRA+ and IGRA- participants had normal chest x-rays. Blood was collected from individuals with active TB within the first 7 days of TB treatment, which was provided according to Kenyan national health guidelines. Helminth infection was determined using standard Kato-Katz microscopy. Briefly, two thick Kato-Katz smears were prepared from stool samples collected on two separate days. Slides were analyzed by experienced lab technicians who recorded the presence of SM eggs as well as the number of eggs counted. Participants were excluded if eggs belonging to other helminth species including *Ascaris lumbricoides*, *Trichuris trichuria*, and hookworm were identified. Other exclusion criteria included: pregnancy, hemoglobin value of <7.0 g/dl, HIV infection, and positive rapid malaria test.

Individuals in Atlanta were enrolled as a naïve control group for both SM and Mtb infection. All individuals were based in the US and had not been vaccinated with BCG. Serologic testing was performed for antibodies against SM eggs as previously described [31]. All US participants in the naïve control group were negative for antibodies to schistosome eggs.

### Sample collection

Blood was collected in sodium heparin Vacutainer CPT Mononuclear Cell Preparation Tubes (BD Biosciences). PBMC were isolated by density centrifugation, cryopreserved in freezing medium (50% RPMI 1640 + 40% heat-inactivated fetal calf serum [FCS] + 10% DMSO), and stored in LN_2_ until use.

### Ethics statement

This study was conducted in accordance with the principles expressed in the Declaration of Helsinki. All participants gave written informed consent for the study, which was approved by the KEMRI Scientific and Ethics Review Unit and the Emory University Institutional Review Board.

### Antigens

This study utilized crude antigen extracts from two distinct stages of the SM life cycle: soluble egg antigen (SEA, 20 μg/ml) and soluble worm antigen preparation (SWAP, 2.5 μg/ml). SEA and SWAP were produced at the U.S. Centers for Disease Control. The positive control for the overnight intracellular cytokine staining assay was phorbol 12-myristate 13-acetate (PMA, 50 ng/ml, Adipogen) and ionomycin (1 μg/ml, Cayman Chemical). Staphylococcal enterotoxin B (SEB; 1 μg/ml, Toxin Technology, Inc.) was used as a positive control for proliferation in the 5-day proliferation assay. During the last 5 hours of the proliferation assay, PMA and ionomycin were used to induce cytokine production.

### Antibodies

The following human monoclonal fluorescently-conjugated antibodies were used in this study: anti-CD3 BV605 (clone OKT-3), anti-CD4 BV570 (clone RPA-T4), anti-TNFα Alexa Flour 647 (clone Mab11), and anti-IL-4 PE-Dazzle594 (clone MP4-25D2), all from BioLegend; anti-CD4 BV786 (clone SK3), anti-CD8 PerCP-Cy5.5 (clone SK-1), anti-TCR γδ BV480 (clone 11f2), and anti-IFNγ Alexa Fluor 700 (clone B27), all from BD Biosciences; and anti-IL-13 FITC (clone 85BRD) from eBiosciences.

### PBMC overnight intracellular cytokine staining (ICS) assay

Cryopreserved PBMC were thawed in a 37°C water bath and immediately added to RPMI 1640 (Cellgro) containing deoxyribonuclease I (DNase, 10 μg/ml, Sigma-Aldrich). Cells were washed in RPMI twice and then suspended in R10 media (RPMI 1640 supplemented with 10% heat-inactivated fetal calf serum [FCS], 100 U/ml penicillin, 100 μg/ml streptomycin, and 2 mM L-glutamine). Cells were rested for a minimum of 3 hours at 37°C and 5% CO_2_ before the addition of antigens (described above). Cells incubated in R10 media alone served as a negative control. Brefeldin A (10 μg/ml; Sigma-Aldrich) and monensin (1x, BioLegend) were added for the last 15 hours of an 18 hour incubation.

### PBMC proliferation assay

Cryopreserved PBMC were thawed, washed in PBS containing deoxyribonuclease I (DNase, 10 μg/ml, Sigma-Aldrich). Cells were washed in PBS twice and then labeled with 0.5 μg/ml CellTrace Oregon Green 488 carboxylic acid diacetate, succinimidyl ester (OG; Life Technologies). Cells were washed once more with PBS and resuspended in R10 media containing recombinant human IL-2 (10 units/ml, obtained through the NIH AIDS Reagent Program, Division of AIDS, NIAID, NIH) [32]. Cells were plated in 96-well plates and incubated for 5 days in a 37°C incubator with 5% CO_2_. On day 5, with the exception of the negative control (wells containing cells in media alone), cells were re-stimulated with PMA and ionomycin (described above) and treated with brefeldin A (10 μg/ml; Sigma-Aldrich) and monensin (1x, BioLegend) for 5 hours at 37°C to determine the cytokine capacity of proliferating T cells.

### Antibody staining and flow cytometry

Following stimulation, cells were washed with PBS and stained with the Fixable Viability Dye Zombie Near-IR (BioLegend) for 15 minutes at room temperature. Samples were then surface stained for 30 minutes at room temperature. For the ICS assay this included: anti-CD3 BV605, anti-CD4 BV570, and anti-CD8 PerCP-Cy5.5. For the proliferation assay: anti-CD3 BV605, anti-CD4 BV786, anti-CD8 PerCP-Cy5.5, and anti-TCR γδ BV480. Following the surface stain, cells were fixed and permeabilized on ice for 1 hour using the FoxP3 Transcription Staining Buffer Set (eBioscience). Cells were then stained for intracellular markers on ice for 40 min. For the ICS assay this included: anti-IFNγ Alexa Fluor 700, anti-TNFα Alexa Flour 647, anti-IL-4 PE-Dazzle594, and anti-IL-13 FITC. For the proliferation assay this included: anti-IFNγ Alexa Fluor 700, anti-TNFα Alexa Flour 647, and anti-IL-4 PE-Dazzle594. Finally, cells were washed in permeabilization buffer and resuspended in PBS. Samples were acquired using a BD LSR II flow cytometer. 6 peak Rainbow Calibration Particles (BioLegend) were used to standardize instrument settings.

### Data analysis

Flow cytometry data were analyzed using FlowJo version 9.6.4 (BD). Compensation was calculated using single-stained anti-mouse Ig,κ CompBeads (BD Biosciences). Single cells were gated by plotting forward scatter-area versus forward scatter-height; lymphocytes were gated based on morphological characteristics. Viable cells were defined as Zombie Near-IR^lo^ cells. In the overnight assay, CD4 T cells were defined as CD3+CD4+CD8- lymphocytes, CD8 T cells were defined as CD3+CD4-CD8+ lymphocytes, and a third population of T cells, referred to as CD4-CD8- T cells, were defined as CD3+CD4-CD8- lymphocytes. In the proliferation assay, CD4 T cells were defined as CD3+CD4+CD8-γδ- lymphocytes, CD8 T cells were defined as CD3+CD4-CD8+γδ- lymphocytes, and γδ T cells were defined as CD3+CD4-CD8-γδ+ lymphocytes. Antigen-specific T cell populations were defined as cells producing cytokines (IFNγ, TNFα, IL-4, or IL-13) after stimulation with antigen. Proliferating cells were defined as those with low expression of the cytosolic dye Oregon Green (OG^lo^). The flow cytometry gating strategies are indicated in S1 Fig. Responses in the proliferation assay were evaluated using the mixture models for single-cell assays (MIMOSA) method to determine positivity using a Markov Chain Monte Carlo algorithm with a prior of 1% [33]. Samples with a probability of response >70% and a false discovery rate (FDR/q-value) <3% were considered positive. Cytokine production from proliferating T cells was restricted to individuals who met the above criteria for a positive response.

### COMPASS analysis of flow cytometry data

Cell counts were analyzed using the COMbinatorial Polyfunctionality Analysis of Antigen-Specific T cell Subsets (COMPASS) algorithm as described previously [34]. COMPASS uses a Bayesian computational framework to identify antigen-specific changes across all observable functional T cell subsets without the need to limit the analysis to specific cytokine combinations. Each analysis was therefore unbiased and considered all 16 cytokine combinations, across each of three T cell subsets (CD4, CD8, and CD4-CD8-) to both SM antigens. For a given participant, COMPASS was also used to compute a functionality score and a polyfunctionality score. A functionality score summarizes the breadth of the cytokine repertoire, taking into account the magnitude of the cytokine response. It is defined as the antigen-specific cytokine subsets detected as a proportion of all possible cytokine subsets. A polyfunctionality score summarizes the diversity of the cytokine repertoire by weighing the different subsets observed based on the number of cytokines in that subset.

### Statistical analysis

R programming software was used to perform all statistical analyses. Differences between SEA and SWAP responses within each infection group or cell type were evaluated using a non-parametric Mann-Whitney test. Differences between three or more groups were evaluated using a non-parametric Kruskal-Wallis test and corrected for multiple pairwise comparisons using the Nemenyi method. P-values < 0.05 were considered significant. Graphs were created using the R package ggplot2 and statistics were performed using the stats and PMCMRplus package.

## Results

### Study participants

Participants were recruited and enrolled in Atlanta, GA, US and Kisumu, Kenya. All participants from Atlanta were seronegative for SEA-specific antibodies and served as naïve controls (N) for SM infection. Participants from Kenya were SM+ and categorized into three groups based on their Mtb infection status: IGRA-, IGRA+, and TB (Table 1). Participants in the IGRA- group were younger than the other participant groups. In addition, there were more females in the N and IGRA- groups than IGRA+ and TB groups. The median egg burden in the IGRA+ group is classified as a moderate intensity infection, whereas the IGRA- and TB groups both had light intensity infections as defined by the WHO [35].

**Table 1:** Characteristics of study participants.

	Naïve (N)	IGRA-	IGRA+	Active TB Disease (TB)	
	n = 12	n = 13	n = 24	n = 16	p-value
SM Status	Serology-	Egg+	Egg+	Egg+	
Age (years)^a^ [IQR]	42 [33–43]	25 [21–32]	34 [25–38]	40 [26–45]	0.005
sex: (%F)	75%	77%	42%	25%	0.01
(%M)	25%	23%	58%	75%	
SM eggs/gram^a^ [IQR]	ND^b^	36 [12–120]	150 [36–333]	48 [21–87]	0.077
QFT IU/ml^a^ [IQR]	ND^b^	0.00 [0.00–0.14]	9.11 [5.36–9.54]	ND ^b^	

^a^ Value denotes median

^b^ ND, not done

IQR, interquartile range

### T cell cytokine production is higher to SWAP than to SEA, irrespective of Mtb and SM infection status

**Fig 1:**
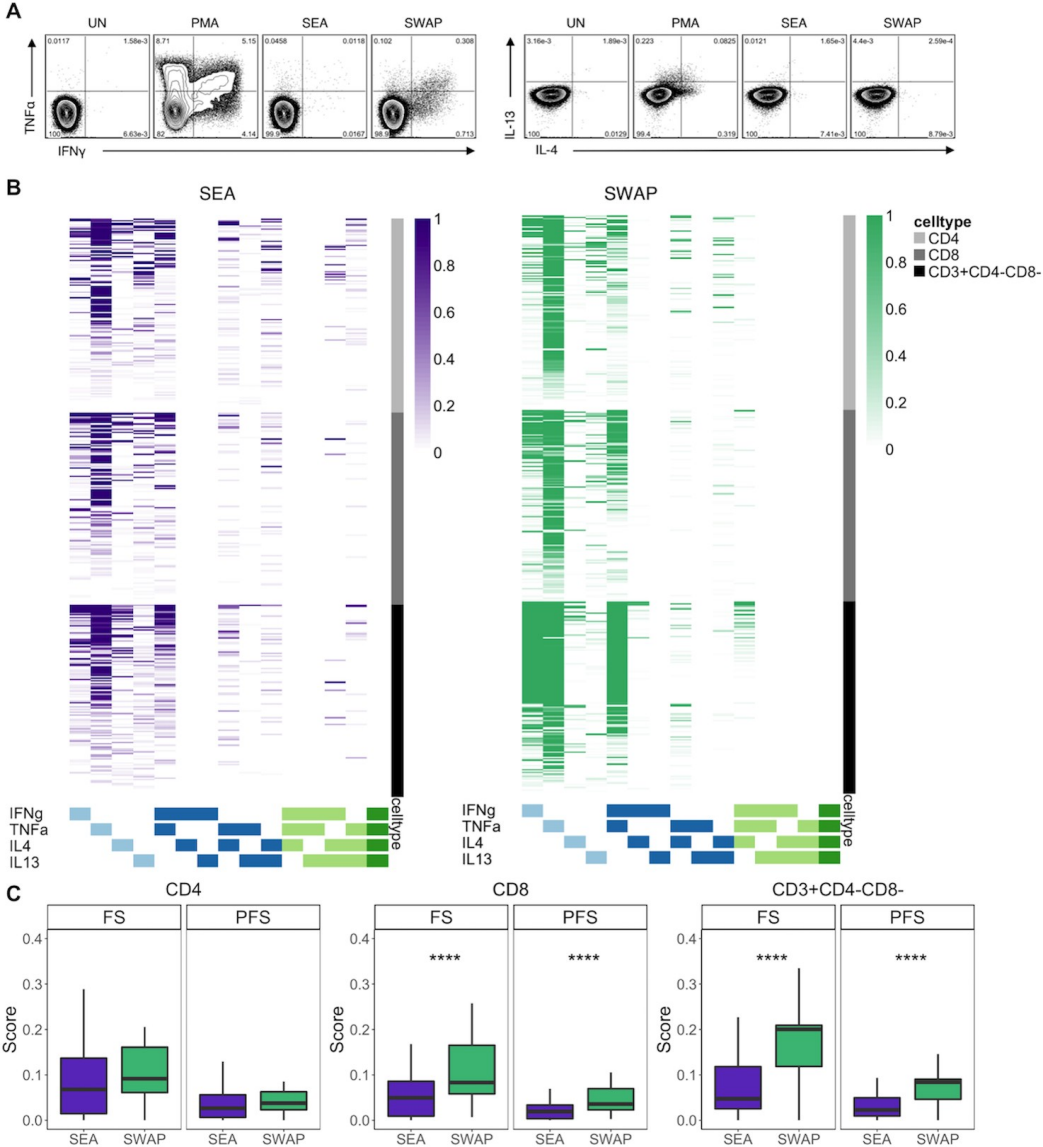
SWAP cytokine responses are higher than SEA cytokine responses among CD8 and CD3+CD4-CD8- T cell subsets. PBMC samples obtained from individuals in each of four groups defined by Mtb and SM infection status (N, n = 12; IGRA-, n = 12; IGRA+, n = 23; TB, n = 15) were incubated for 18 h in media alone (negative control) or stimulated with SEA, SWAP or PMA and ionomycin (positive control). Intracellular expression of IFNγ, TNFα, IL-4, and IL-13 was measured by flow cytometry. (**A**) Representative flow cytometry data from a Naïve individual. Plots show cells gated on live CD3+ lymphocytes from the unstimulated (UN), PMA, SEA, and SWAP stimulated conditions. (**B**) ICS data were analyzed using COMPASS and the results from each cytokine subset are displayed as a heatmap of posterior probabilities of antigen specificity. Rows represent study subjects and columns represent cytokine combinations. The intensity of shading represents the probability of detecting a response above background on a scale of 0–1. (**C**) Subject-specific COMPASS results were summarized for 63 individuals using the functionality score (FS) and polyfunctionality scores (PFS). Scores from CD4, CD8, and CD3+CD4-CD8- T cell subsets were aggregated across all groups. Boxes represent the median and interquartile ranges; whiskers represent the 1.5*IQR. Differences in the scores of each T cell population between SEA and SWAP were assessed using a Mann-Whitney U test. **** p<0.0001.

Human SM-specific CD8 T cell responses and non-classical (CD4-CD8-) T cell responses have not been thoroughly investigated. To characterize the T cell repertoire to SM antigens from distinct stages of the SM life cycle, we stimulated PBMC overnight with media alone (negative control), PMA and ionomycin (positive control), SEA, and SWAP. We then performed intracellular cytokine staining (ICS) for the type 1 cytokines IFNγ and TNFα, as well as the type 2 cytokines IL-4 and IL-13 for analysis by flow cytometry (Fig 1A). Total cytokine frequencies were evaluated in CD4, CD8, and CD4-CD8- T cells and were higher following stimulation with SWAP than SEA (S2 Fig). To analyze these data in an unbiased manner, we utilized the statistical package COMPASS which allowed us to evaluate all possible cytokine combinations across all three T cell subsets to both SEA and SWAP (Fig 1B). The dominant cytokine producing subset in both the SEA and SWAP stimulation was TNFα single positive T cells. More individuals had IL-4+ T cell responses in the SEA condition than the SWAP condition. More individuals had TNFα+IFNγ+ T cells in the SWAP condition than in the SEA condition, predominantly amongst CD4-CD8- T cells. To confirm that these observations were specific to the SWAP stimulation, the same analysis was peformed on T cells following stimulation with PMA and ionomycin. Overall, the cytokine response to PMA and ionomycin was very robust, with CD4 T cells having an overall higher functionality score (FS) than CD8 and CD4-CD8- cells (S3 Fig). These scores are driven by production of IFNγ and TNFα in all cell types (S4 Fig).

We also utilized COMPASS to generate summary scores for both the overall FS, as well as the polyfunctionality score (PFS) of each sample (Fig 1C). In CD8 and CD4-CD8- T cells, SWAP responses were higher than SEA responses with regard to both FS and PFS. Upon stratification by Mtb and SM status, this held true in the IGRA-, IGRA+, and TB groups, but not the N group (S5 Fig). Together these data indicate that T cell responses to SWAP are more robust than T cell responses to SEA.

### T cell responses to SWAP are dominated by non-classical T cells

**Fig 2:**
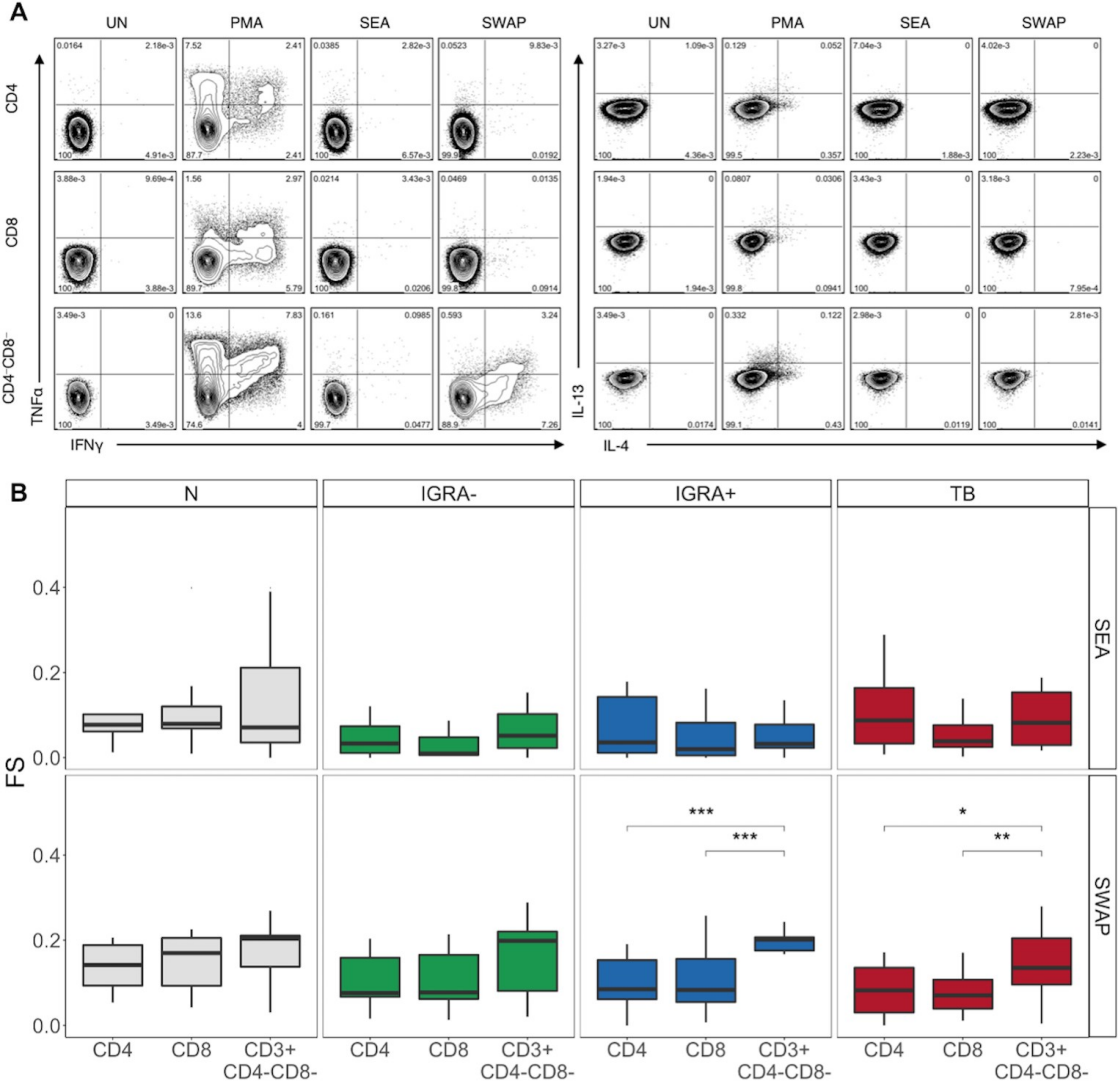
CD3+CD4-CD8- T cells have a greater functional response to SWAP than CD4 and CD8 T cells in IGRA+ and TB individuals. PBMC from individuals in each group were stimulated and analyzed by flow cytometry as described in Fig 1. Intracellular expression of IFNγ, TNFα, IL-4, and IL-13 was measured by flow cytometry. **(A)** Representative flow cytometry data from a Naïve individual. Plots are shown gated on live CD3+CD4+CD8-, CD3+CD4-CD8+, and CD3+CD4-CD8- lymphocytes from the unstimulated (UN), PMA, SEA, and SWAP stimulated condition. **(B)** COMPASS functionality scores among each T cell subset. Boxes represent the median and interquartile ranges; whiskers represent the 1.5*IQR. Differences in the scores of each T cell subset were assessed using a Kruskal-Wallis test with Nemenyi correction for multiple pairwise comparisons. *** p<0.001; ** p< 0.01; * p< 0.05.

We next utilized COMPASS to determine which T cell subsets had the strongest response to SEA and SWAP (Fig 2A). SEA-specific T cell FS were equivalent between CD4, CD8, and CD4-CD8- subsets in all four groups (Fig 2B). In contrast, SWAP-reactive FS were higher in CD4-CD8- T cells, compared to both CD4 and CD8 T cells in the IGRA+ and TB groups (Fig 2B). This was also true for SWAP-reactive PFS (S6 Fig). These data indicate that while there is no dominant SEA-specific T cell type, CD4-CD8- T cells are the dominant T cell population responding to SWAP.

Since these differences were only observed in some groups, we next evaluated whether Mtb or SM infection affected T cell FS. Importantly, the only differences in FS and PFS scores between Mtb groups were in CD8 T cells (S7 Fig). SEA-specific CD8 T cell scores were higher in N compared to IGRA-. SWAP-reactive CD8 T cell scores were higher in N compared to TB individuals. These data indicate that SM but not Mtb infection modestly alters SM-specific CD8 T cell responses.

### SWAP induces more robust T cell proliferation than SEA

**Fig 3:**
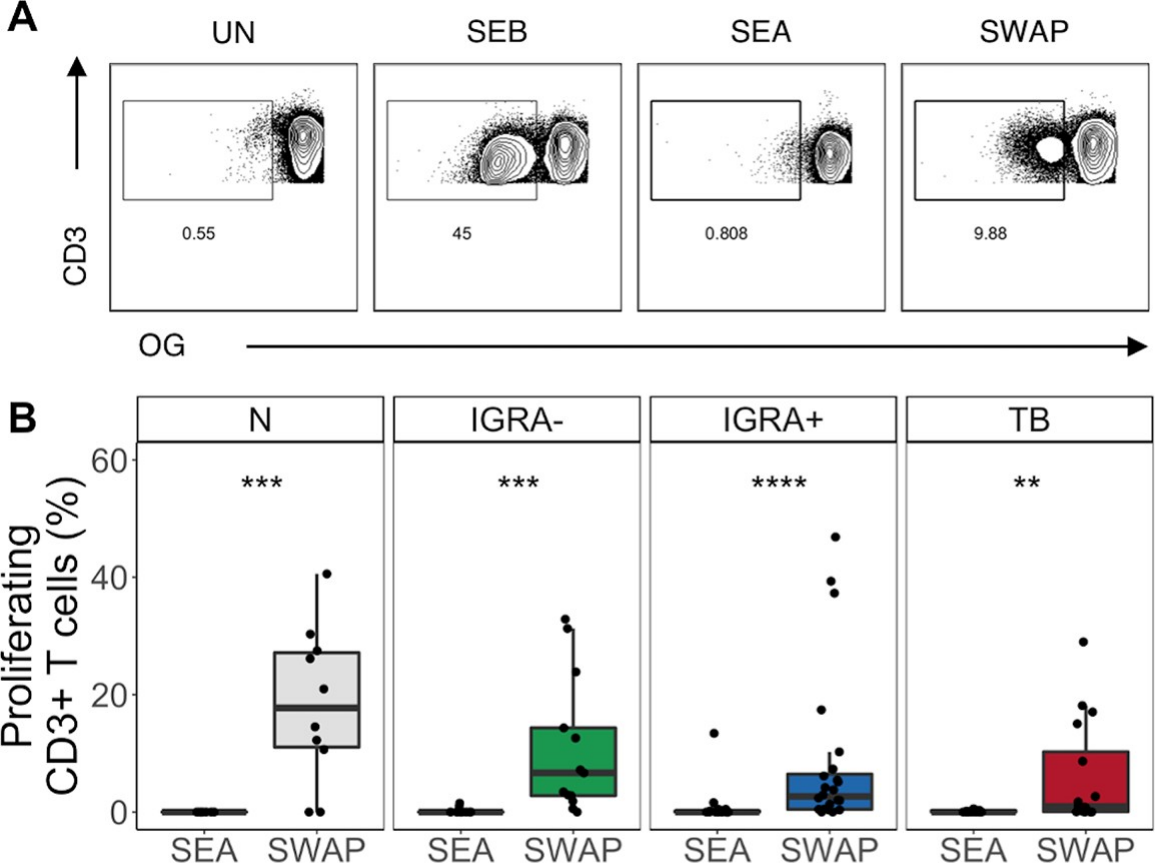
Higher frequencies of T cells proliferate in response to SWAP than in response to SEA, independent of Mtb and SM infection status. Proliferation assays were performed using PBMC samples obtained from individuals in each of four groups defined by Mtb and SM infection status (N, n = 10; IGRA-, n = 13; IGRA+, n = 24; TB, n = 16). Cells were labeled with Oregon Green (OG) and incubated for 5 days under the following conditions: media alone (negative control), SEB (positive control), SEA or SWAP. **(A)** Representative flow cytometry data from an IGRA+ individual. Plots show cells gated on live CD3+ lymphocytes. **(B)** Frequency of OG^lo^ (proliferating) T cells to SEA and SWAP. Data are shown after subtraction of background proliferation in the unstimulated negative control condition. Boxes represent the median and interquartile ranges; whiskers represent the 1.5*IQR. Differences in the frequency of proliferating T cells between SEA and SWAP were assessed using a Mann-Whitney U test. **** p<0.0001; *** p<0.001; ** p< 0.01.

To further characterize the SM T cell repertoire, we next performed a proliferation assay. PBMC from each group were labeled with the cytosolic dye Oregon Green (OG) and incubated for 5 days with media alone (negative control), SEB (positive control), SEA or SWAP. We then measured proliferation of total T cells via flow cytometry (Fig 3A). Similar to what was observed in the overnight ICS assay, the CD3+ T cell proliferative response to SWAP was higher than to SEA in all groups (Fig 3B). These data indicate that T cells proliferate more robustly to SWAP than to SEA.

### γδ T cells are the dominant population of T cells responding to SWAP

**Fig 4:**
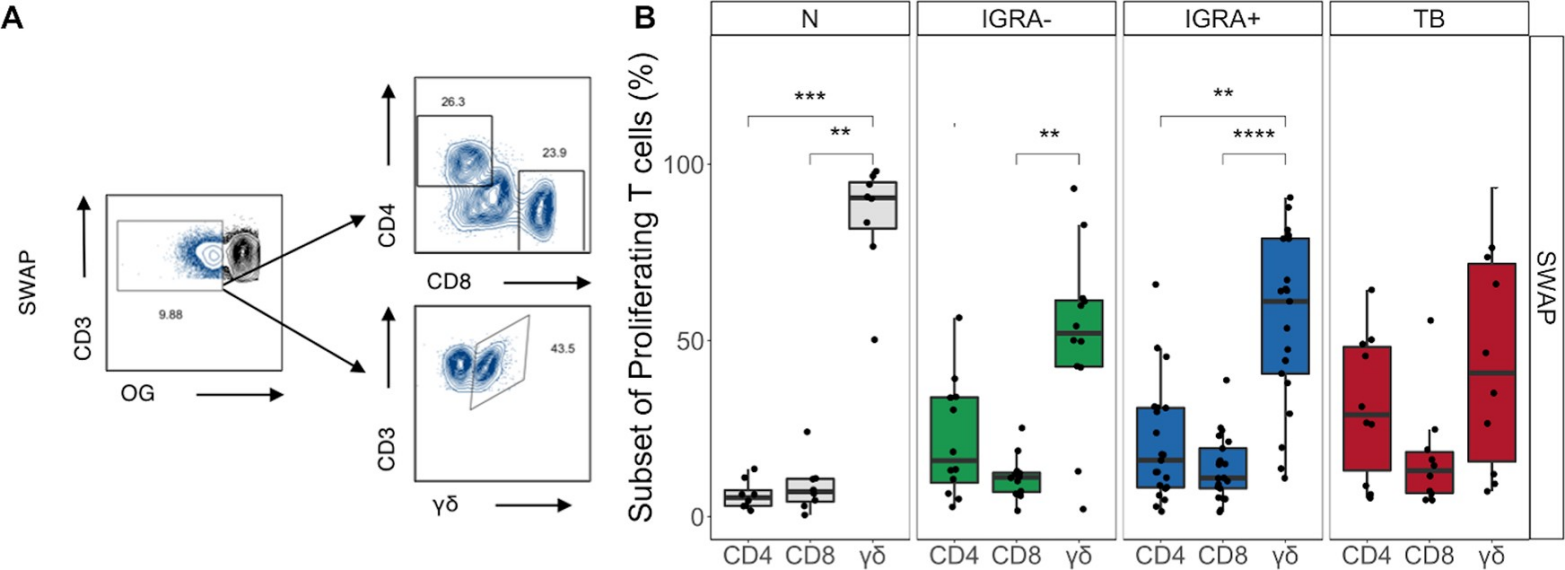
Proliferating SWAP-reactive T cells are predominantly γδ T cells. Proliferating CD3+ T cells from the SWAP conditions were evaluated by flow cytometry for expression of CD4, CD8, and γδ. **(A)** Representative flow cytometry data from an IGRA+ individual. Plots show the expression of CD4, CD8, and γδ on cells gated on live OG^lo^ CD3+ lymphocytes. **(B)** The proportions of SWAP-reactive proliferating T cells that are CD4, CD8, and γδ T cells were evaluated in each Mtb and SM infection group. Boxes represent the median and interquartile ranges; whiskers represent the 1.5*IQR. Differences in the frequency of each T cell population was assessed using a Kruskal-Wallis test with Nemenyi correction for multiple pairwise comparisons. **** p<0.0001; *** p<0.001; ** p< 0.01.

Peripheral γδ T cells expand in the blood of people infected with a variety of pathogens [22,24]. Since we observed a robust response of CD4-CD8- T cells to SWAP during the overnight ICS assay, we included an antibody to detect γδ T cell receptor expression in the proliferation assay. We used MIMOSA (see Materials and Methods) to determine which samples had a positive proliferative response. These samples were evaluated for the distribution of CD4, CD8 and γδ T cells using flow cytometry (Fig 4A). Amongst SWAP-reactive proliferating T cells, γδ T cells were the dominant subset in the N, IGRA-, and IGRA+ groups (Fig 4B). Due to the low frequency of SEA-specific proliferating T cells across all groups, we were not able to evaluate which T cell subsets proliferated in response to SEA.

### TB modifies the SWAP-reactive γδ T cell response

**Fig 5:**
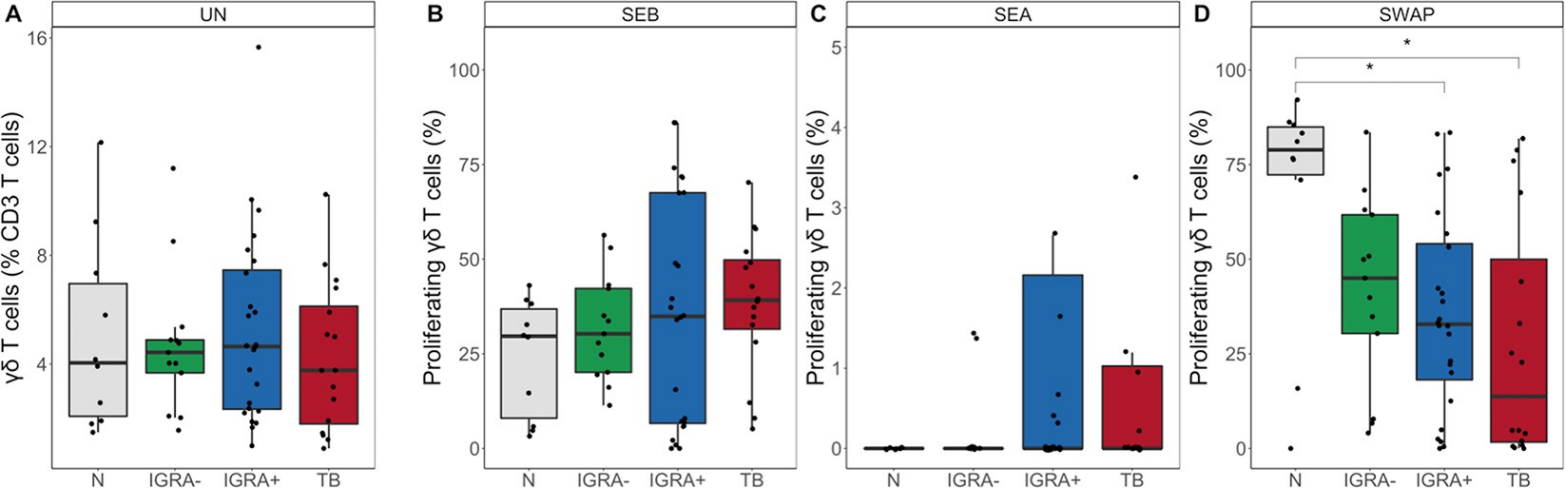
SWAP-reactive γδ T cells have lower proliferative capacity in individuals with IGRA+ and active TB. Proliferation assays were performed as described in Fig 3. **(A)** Frequency of total γδ T cells in the unstimulated (UN) condition (N, n = 10; IGRA-, n = 13; IGRA+, n = 24; TB, n = 16). **(B-D)** Frequency of proliferating (OG^lo^) γδ T cells to SEB **(B),** SEA **(C)** and SWAP **(D)**. Proliferation data are shown after subtraction of background proliferation in the UN condition. Boxes represent the median and interquartile ranges; whiskers represent the 1.5*IQR. Differences in the frequency of each proliferating γδ T cell population between groups were assessed using a Kruskal-Wallis test with Nemenyi correction for multiple pairwise comparisons. * p< 0.05.

We next determined whether Mtb infection status modified the γδ T cell response to SWAP. We first measured the frequency of γδ T cells at day 5 in the unstimulated condition, which did not differ between the four groups (Fig 5A). We next evaluated the frequency of proliferating γδ T cells in the SEB, SEA, and SWAP conditions. The frequency of proliferating cells did not differ aross the groups in the SEB or SEA condition (Fig 5B and 5C). However, SWAP-reactive γδ T cell proliferation was significantly lower in the IGRA+ and TB groups compared to the N group (Fig 5D). Together, these data indicate that there is not an inherent defect in γδ T cell numbers or proliferative capacity due to Mtb infection. We next evaluated whether the difference in proliferative capacity was specific to γδ T cells. Importantly, the proliferative capacity of CD4+ T cells and total CD3+ T cells in response to SEB, SEA, and SWAP did not differ between the groups (S8 and S9 Figs). These data indicate that Mtb infection status specifically impacts the proliferative capacity of γδ T cells to SWAP and not other antigenic stimuli.

**Fig 6:**
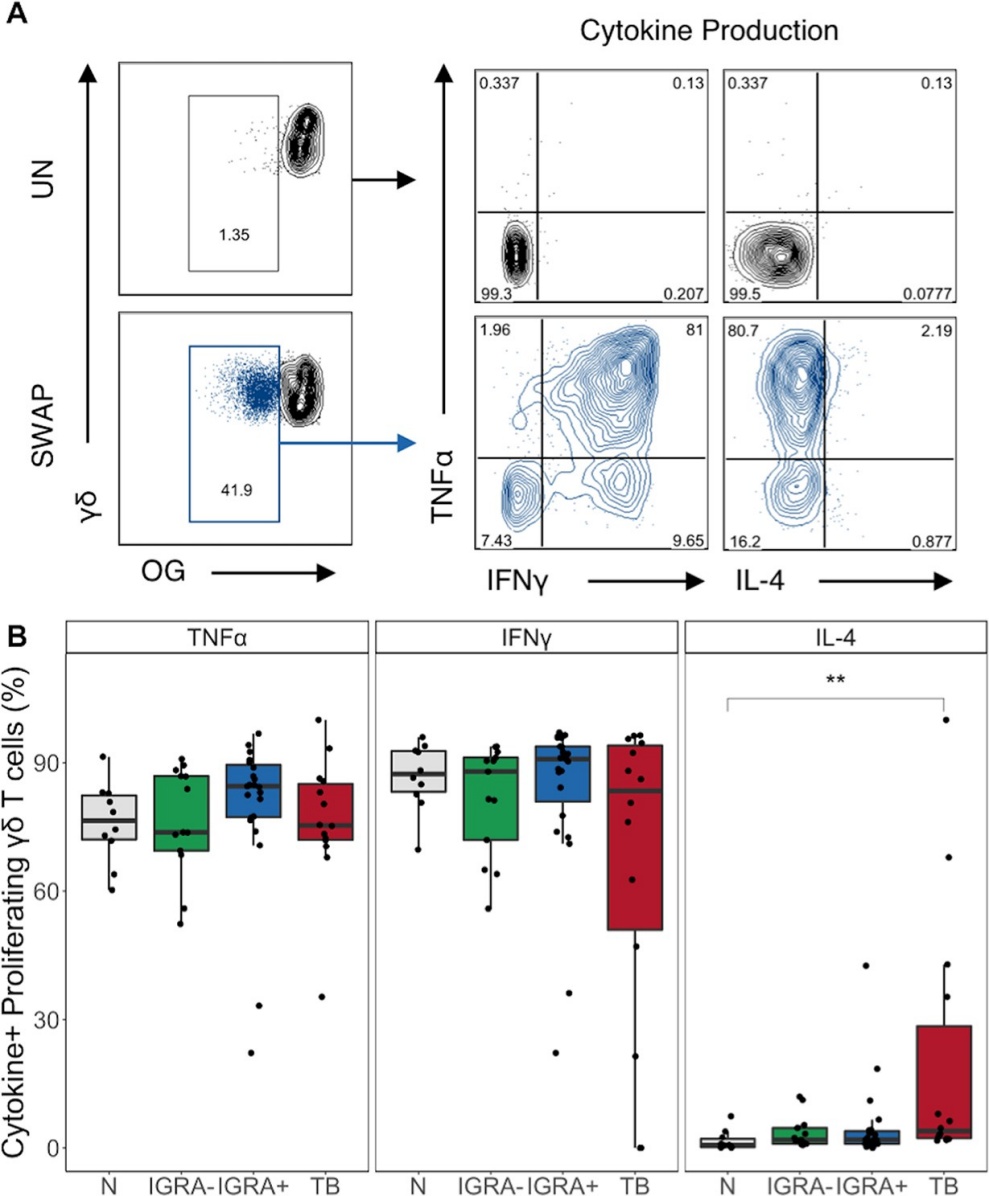
Individuals with active TB have higher frequencies of IL-4 producing SWAP-reactive γδ T cells. On day 5 of the proliferation assay PBMC were restimulated with PMA and ionomycin for 5 hours to induce cytokine production. Samples meeting the criteria for a positive proliferative response (see Materials and Methods) were evaluated for cytokine expression (N, n = 8; IGRA-, n = 12; IGRA+, n = 21; TB, n = 10). **(A)** Representative flow plots from an IGRA+ individual. Unstimulated samples (upper) show cytokine production of cells gated on live CD3+CD4-CD8-γδ+ lymphocytes. SWAP-stimulated samples (lower) show cytokine production and phenotypes of cells gated on live OG^lo^CD3+CD4-CD8-γδ+ lymphocytes. **(B)** Frequency of cytokine+ cells amongst proliferating γδ T cells. Boxes represent the median and interquartile ranges; whiskers represent the 1.5*IQR. Differences in the frequency of each cytokine+ γδ T cell population between groups were assessed using a Kruskal-Wallis test with Nemenyi correction for multiple pairwise comparisons. ** p< 0.01.

We next evaluated the cytokine profiles of proliferating SWAP-reactive γδ T cells. On day 5 of the proliferation assay, we restimulated cells using PMA and ionomycin for 5 hours and performed intracellular cytokine staining for analysis by flow cytometry (Fig 6A). Similar to the overnight assay, high frequencies of SWAP-reactive γδ T cells produce TNFα and IFNγ, while very low frequencies produce IL-4. While TNFα and IFNγ production capacity did not vary between groups, there were higher frequencies of IL-4 producing SWAP-reactive γδ T cells in the active TB group compared to the N group. Taken together, these data demonstrate that not only are γδ T cells impaired in Mtb-infected individuals with regard to proliferative capacity, but they also have skewed cytokine profiles in individuals with active TB.

## Discussion

SM has a complex life cycle which involves multiple distinct morphological stages and immunological phases in the host [5]. In mice, this is largely characterized by a type 1 CD4 T cell response to the larval stage which shifts to a type 2 CD4 T cell response upon egg laying (5, 9). However, less is known about the phenotype of the T cell response to the adult stage of the worm and which T cell subsets contribute to the overall immune response to SM. In this study, we sought to characterize the T cell repertoire to antigens from different stages of the SM life cycle, as well as determine the impact of Mtb infection on SM-specific T cell responses. We determined that the T cell response to SWAP was significantly more robust than the T cell response to SEA. We also found that the dominant T cell subset to respond to SWAP consisted of γδ T cells. Lastly, we determined that Mtb infection impaired the ability of γδ T cells to proliferate in response to SWAP and altered the cytokine profile of these T cells in patients with active TB disease.

Our data indicate that T cell responses are higher to SWAP than to SEA with regard to both cytokine production and proliferative capacity. This phenomenon is particularly evident in the CD8 and non-classical T cell compartments of SM infected individuals without active TB. While SEA is strongly immunogenic during acute infection and experimental models, it is generally accepted that in endemic settings, the immune response to egg antigens are regulated to limit immunopathology [6,36,37]. It is also generally accepted that SWAP responses are maintained during chronic infection. This is largely based on the fact that antibody responses to worm stage antigens rise during infection and stay high in chronically infected individuals [36,38]. The data supporting the preservation of SWAP responses has not, however, directly addressed the role of T cells. In studies measuring PBMC proliferation after stimulation with SEA or SWAP, the proportion of acutely infected individuals who respond to SEA is much higher than the proportion of chronically infected individuals. In contrast, the proportion of individuals who respond to SWAP is consistent between groups of acute and chronically infected individuals [37]. In addition, PBMC from chronically infected individuals show higher cytokine production and phosphorylation of T cell signaling molecules following stimulation with SWAP than SEA [39,40]. It should be noted that while our results highlight a robust TH1 cytokine response to SWAP, previous studies in the region have found that PBMC and whole blood cultures stimulated with SWAP are capable of producing both TH1 and TH2 cytokines [41–43]. This is likely due do differences in methodology, particularly the use of flow cytometry, which may not be able to robustly detect TH2 cytokine production. These studies and our data suggest that in chronically infected individuals, the T cell response to SWAP is maintained while the T cell response to SEA is not.

One strength of our study is that we did not limit our analysis to CD4 T cells, which allowed us to detect a strong γδ T cell response to SWAP antigen. γδ T cells are elevated in the blood of individuals with acute schistosomiasis, however this analysis was not done in an antigen-specific fashion [25]. Here we provide evidence that γδ T cells respond to SM in an antigen specific manner in that they are activated by SWAP but not by SEA. Furthermore, SWAP-reactive responses were characterized by high production of TNFα and IFNγ. In other infections, type 1 cytokine producing γδ T cells link the innate and adaptive arms of the immune system [23]. Thus, our study has uncovered a potential novel role for SM-specific γδ T cells during infection which should be investigated further.

Our findings also provide strong evidence that Mtb infection alters the response of γδ T cells to SWAP. Indeed, we observe decreased proliferative capacity of SWAP-reactive γδ T cells in individuals infected with Mtb. Furthermore, in individuals with active TB disease, we observed an increase in IL-4 producing γδ T cells. Previous work on Mtb-specific γδ T cells has shown a decrease in blood γδ T cells during active TB disease and has suggested that Mtb infection induces chemokines in the lung which recruit blood dwelling γδ T cells into the lung [44]. It is possible that SM-specific γδ T cells respond to similar signals and are therefore depleted from the blood, leading to the diminished responsiveness observed in this study.

The interpretation of our study is limited by enrollment of study participants at a single time point. As such, we are not able to determine the order of infection of SM and Mtb, or the duration of current SM infection. In addition, due to sample availability we were not able to evaluate previous infection history. These factors may play a role, not only in how T cells respond to SM antigens from different stages of the SM life cycle, but also in the potential for Mtb to impair these responses. This is particularly relevant since in western Kenya children as young as 3 years old have been shown to be infected with SM [45]. SM infections reach a prevalence of 60% by the time children are 11–13 years old [46]. As such, there is a high probability that study participants have had previous exposure to SM. We have tried to account for previous SM infection by including a naïve control group of U.S. adults, who are seronegative for SM antibodies indicating no previous exposure. These individuals, however, are U.S. residents and therefore differ in other unmeasured ways from the Kenyan participants.

In addition, the definitions used for Mtb infection outcomes may influence the interpretation of our results. We have used the labels IGRA+ and IGRA- throughout this study since there is currently no gold standard for diagnosing IGRA+ [47], however IGRA assays are imperfect. First and foremost, a positive IGRA does not differentiate IGRA+ from subclinical or active TB since the assay is based on an immune response to Mtb antigens [48]. It has also been demonstrated that even IGRA negative individuals can still go on to develop TB [49]. Lastly, there is significant within-subject variability that can lead to discordant test results during serial testing [47].

It should be noted that we were not able to characterize the full T cell repertoire in this study. Notably we did not include markers to identify mucosally associated invariant T cells (MAIT cells) or invariant natural killer T cells (iNKT cells). Because both of these non-classical T cell subsets are CD4-CD8-, we cannot conclude that all of the CD4-CD8- T cells observed in our overnight assay were γδ T cells. However, the robust responses of γδ T cells in the proliferation assay suggest that the cytokine responses observed in the overnight assay were due to γδ T cells. Furthermore iNKT cells constitute a very small portion of T cells in the blood (~0.5%) [50] and there are no currently published studies that suggest that MAIT cells are involved in the immune response to helminth infections. Indeed, it is believed that MAIT cells respond specifically to metabolites derived from bacterial and fungal vitamin B synthesis pathways [51–53]. Similarly, we were unable to confirm that cells defined as CD8 T cells in the overnight assay did not include γδ T cells. A subset of γδ T cells, termed intraepithelial lymphocytes, express CD8, however these are tissue-resident and therefore not likely present in our PBMC samples [54,55]. Lastly we were not able to determine the v-chain usage of the SWAP responding γδ T cells. In humans γδ T cells expressing the Vδ2 and the Vγ2/Vγ9 (used interchangeably in the literature) chain are the dominant subset in the blood [19–21,56,57]. As such, we assume these are Vγ2Vδ2 cells. This is of particular interest since Vγ2Vδ2 T cells posess memory-like properties in murine models of Listeria and staphylococcal infection and non-human primate (NHP) models of BCG vaccination and Mtb infection [23,58]. Furthermore, in NHP models of Mtb infection, priming γδ T cells using the γδ specific ligand (E)-4-hydroxy-3-methyl-but-2-enyl pyrophosphate (HMBPP) protected animals against Mtb challenge [59].

Lastly, because SWAP is a complex mixture of antigens, we were not able to determine which specific antigen is recognized by γδ T cells. Previous literature has demonstrated that low molecular weight fractions of SWAP are responsible for inducing IFNγ production, however the specific antigens present in this fraction are still unknown [60]. Peripheral blood γδ T cells respond to phosphoantigens from a variety of microbes [56]. Two known γδ phosphoantigens, HMBPP and isopentenyl pyrophosphate (IPP), are both intermediates of isoprenoid synthesis pathways [26]. Genetic analysis demonstrates that SM possesses genes for the enzymes involved in the mevalonate pathway of isoprenoid synthesis and therefore may produce IPP during the adult stage of the life cycle [61,62]. Furthermore, the drug mevinolin, which targets isoprenoid synthesis impairs egg production from schistosome worms, suggesting it is actively produced by adult worms [63]. IPP is therefore a strong candidate ligand for γδ T cells.

In this study, we provide evidence that γδ T cells have the capacity to respond to SM worm antigens by proliferating and producing type 1 cytokines. The immune response to SM is complex and dynamic, but has mostly been characterized by type 2 responses. Indeed, the focus of most helminth immunology has been on type 2 and regulatory responses for their potential applications in other fields. While the contribution of type 1 cytokine producing γδ T cells during SM infection remains unclear, there are many potential roles for these γδ T cells in infection with SM. Studies in mice have shown that early IFNγ production can provide protection against SM infection, however this was directed at cercarial antigen and not adult worms [64]. Furthermore, high IFNγ responses to SEA antigen were associated with protection in a cohort of persistently uninfected individuals from an endemic area in Brazil [65]. Whether type 1 cytokines produced in response to the adult worm can provide similar benefits is unknown, particularly because adult worms are heavily shielded from the immune system by their external tegument [66]. As such, the antigens in SWAP that induce γδ T cell responses may be hidden until the worm dies. It has been hypothesized, however, that these antigens may become exposed following treatment with praziquantel and that SWAP-specific immune responses may synergize with praziquantel to achieve worm death [67]. Robust SWAP-specific responses in PBMC have also been associated with resistance to reinfection [68]. Future studies in mice or NHP would benefit from expanding study of γδ T cells at different stages of infection to determine their function in vivo and their potential in mediating protection from SM.

## Supporting information

S1 Fig Gating strategy for flow cytometry analysis. **(A)** In this sample gating for the overnight ICS assay, cells were first gated for singlets (FSC-H vs. FSC- A) and lymphocytes (SSC-A vs. FSC-A). The lymphocyte gate is further analyzed for their uptake of the Zombie IR Live/Dead stain to determine live versus dead cells and their expression of CD3 (Zombie Near-IR^lo^, CD3+). CD4 and CD8 surface expression is then determined from this gated population. **(B)** In this sample gating for the Proliferation ICS assay, cells were first gated for singlets (FSC-H vs. FSC- A) and lymphocytes (SSC-A vs. FSC-A). The lymphocyte gate is further analyzed for their uptake of the Zombie IR Live/Dead stain to determine live versus dead cells (Zombie Near-IR^lo^). Live cells are then gated for their expression of CD3 (CD3+). CD4, CD8, and γδ surface expression is then determined from this gated population. CD4 T cells were defined as CD3+CD4+CD8- lymphocytes, CD8 T cells were defined as CD3+CD4-CD8+ lymphocytes, and a third population of T cells were defined as CD3+CD4-CD8- lymphocytes.
(PDF)

S2 Fig Cytokine frequencies are higher following SWAP stimulation than SEA stimulation. PBMC from individuals in each group were stimulated and analyzed by flow cytometry as described in Fig 1. Intracellular expression of IFNγ, TNFα, IL-4, and IL-13 was measured by flow cytometry. **(A)** Frequency of total cytokine+ cells within each designated cell type are reported. **(B)** Frequency of each combination of cytokine+ cells using a Boolean gating strategy within each cell type are reported. Boxes represent the median and interquartile ranges; whiskers represent the 1.5*IQR. Differences in the cytokine frequency between SEA and SWAP were assessed using a Mann-Whitney U test. **** p<0.0001; *** p<0.001; ** p< 0.01; * p< 0.05.
(PDF)

S3 Fig CD4 T cells have a greater functional response to PMA/ionomycin than CD8 and CD3+CD4-CD8- T cells. PBMC from individuals in each group were stimulated with PMA and analyzed by flow cytometry as described in Fig 1. Intracellular expression of IFNγ, TNFα, IL-4, and IL-13 was measured by flow cytometry. (**A**) ICS data were analyzed using COMPASS and the results from each cytokine subset are displayed as a heatmap. Rows represent study subjects and columns represent cytokine combinations. The intensity of shading represents the probability of detecting a response above background. (**C**) Subject-specific COMPASS results were summarized for 63 individuals using the functionality and polyfunctionality scores. Scores from CD4, CD8 and CD3+CD4-CD8- T cell subsets were aggregated across all groups. Boxes represent the median and interquartile ranges; whiskers represent the 1.5*IQR. Differences between the scores of each T cell subset were assessed using a Kruskal-Wallis test with Nemenyi correction for multiple pairwise comparisons. *** p<0.001; ** p< 0.01; * p< 0.05.
(PDF)

S4 Fig IFNγ and TNFα are produced in response to PMA and Ionomycin across T cell types. PBMC from individuals in each group were stimulated with PMA and analyzed by flow cytometry as described in Fig 1. Intracellular expression of IFNγ, TNFα, IL-4, and IL-13 was measured by flow cytometry. Frequency of each combination of cytokine+ cells using a Boolean gating strategy within each cell type are reported. Boxes represent the median and interquartile ranges; whiskers represent the 1.5*IQR.
(PDF)

S5 Fig SWAP functionality and polyfunctionality scores are higher than SEA responses in IGRA- and IGRA+ groups. PBMC from individuals in each of four groups defined by Mtb and SM infection status (N, n = 12; IGRA-, n = 12; IGRA+, n = 23; TB, n = 15) were incubated for 18 h in media alone (negative control) or stimulated with SEA or SWAP. Intracellular expression of IFNγ, TNFα, IL-4, and IL-13 was measured by flow cytometry and data were analyzed using COMPASS. **(A-C)** Functionality and polyfunctionality scores for CD4 **(A)**, CD8 **(B)**, and CD3+CD4-CD8- **(C)** T cells. Boxes represent the median and interquartile ranges; whiskers represent the 1.5*IQR. Differences in the scores of each T cell subset were assessed using a Kruskal-Wallis test with Nemenyi correction for multiple pairwise comparisons. **** p<0.0001; *** p<0.001; ** p< 0.01; * p< 0.05.
(PDF)

S6 Fig CD3+CD4-CD8- T cells have a greater polyfunctional response to SWAP than CD4 and CD8 T cells in IGRA+ and TB individuals. PBMC samples obtained from individuals in each of four groups defined by Mtb and SMinfection status (N, n = 12; IGRA-, n = 12; IGRA+, n = 23; TB, n = 15) were incubated for 18 hours in media alone (negative control) or stimulated with SEA or SWAP. Intracellular expression of IFNγ, TNFα, IL-4, and IL-13 was measured by flow cytometry. COMPASS functionality scores among each T cell subset are reported. Boxes represent the median and interquartile ranges; whiskers represent the 1.5*IQR. Differences in the scores of each T cell subset were assessed using a Kruskal-Wallis test with Nemenyi correction for multiple pairwise comparisons. *** p<0.001; ** p< 0.01.
(PDF)

S7 Fig CD8 T cell responses to both SEA and SWAP are higher in Naïve individuals. PBMC samples obtained from individuals in each of four groups defined by Mtb and SM infection status (N, n = 12; IGRA-, n = 12; IGRA+, n = 23; TB, n = 15) were incubated for 18 hours in media alone (negative control) or stimulated with SEA or SWAP. Intracellular expression of IFNγ, TNFα, IL-4, and IL-13 was measured by flow cytometry and data were analyzed using COMPASS. **(A-C)** SEA-specific FS and PFS for CD4 **(A)**, CD8 **(B)**, and CD4-CD8- **(C)** T cells. **(D-F)** SWAP-specific FS and PFS for CD4 **(D)**, CD8 **(E)**, and CD4-CD8- **(F)** T cells. Boxes represent the median and interquartile ranges; whiskers represent the 1.5*IQR. Differences in the scores of each T cell subset were assessed using a Kruskal-Wallis test with Nemenyi correction for multiple pairwise comparisons. ** p< 0.01.
(PDF)

S8 Fig SWAP-reactive CD4 T cell proliferation capacity is equivalent between groups. Proliferation assays were performed as described in Fig 3. **(A)** Frequency of total CD4 T cells in the unstimulated (UN) condition (N, n = 10; IGRA-, n = 13; IGRA+, n = 24; TB, n = 16). **(B-D)** Frequency of proliferating (OG^lo^) CD4 T cells to SEB **(B),** SEA **(C)** and SWAP **(D)**. Proliferation data are shown after subtraction of background proliferation in the UN condition. Boxes represent the median and interquartile ranges; whiskers represent the 1.5*IQR. Differences in the frequency of each proliferating CD4 T cell population between groups were assessed using a Kruskal-Wallis test with Nemenyi correction for multiple pairwise comparisons.
(PDF)

S9 Fig Proliferation of total CD3+ T cells does not differ by Mtb and SM infection status. Proliferation assays were performed using PBMC obtained from individuals in each of four groups defined by Mtb and SM infection status (N, n = 10; IGRA-, n = 13; IGRA+, n = 24; TB, n = 16). Cells were labeled with Oregon Green (OG) and incubated for 5 days under the following conditions: media alone (negative control), SEB (positive control), SEA or SWAP. **(A-C)** Frequency of OG^lo^ (proliferating) CD3+ T cells to SEB **(A)**, SEA **(B)** and SWAP **(C)**. Data are shown after subtraction of background proliferation in the unstimulated negative control condition. Boxes represent the median and interquartile ranges; whiskers represent the 1.5*IQR. Differences in the frequency of each proliferating CD3+ T cell population between groups were assessed using a Kruskal-Wallis test with Nemenyi correction for multiple pairwise comparisons.
(PDF)

## References

[1] Hotez PJ, Brindley PJ, Bethony JM, King CH, Pearce EJ, Jacobson J. Helminth infections: the great neglected tropical diseases. J Clin Invest. 2008 4 1;118(4):1311–21. 10.1172/JCI34261 18382743 PMC2276811

[2] Hotez PJ, Kamath A. Neglected Tropical Diseases in Sub-Saharan Africa: Review of Their Prevalence, Distribution, and Disease Burden. Cappello M, editor. PLoS Negl Trop Dis. 2009 8 25;3(8):e412 10.1371/journal.pntd.0000412 19707588 PMC2727001

[3] van der Werf MJ, de Vlas SJ, Brooker S, Looman CWN, Nagelkerke NJD, Habbema JDF, et al Quantification of clinical morbidity associated with schistosome infection in sub-Saharan Africa. Acta Tropica. 2003 5;86(2–3):125–39. 10.1016/s0001-706x(03)00029-9 12745133

[4] Nelwan ML. Schistosomiasis: Life Cycle, Diagnosis, and Control. Current Therapeutic Research. 2019;91:5–9. 10.1016/j.curtheres.2019.06.001 31372189 PMC6658823

[5] Pearce EJ, MacDonald AS. The immunobiology of schistosomiasis. Nat Rev Immunol. 2002 7;2(7):499–511. 10.1038/nri843 12094224

[6] Colley DG, Secor WE. Immunology of human schistosomiasis. Parasite Immunol. 2014 8;36(8):347–57. 10.1111/pim.12087 25142505 PMC4278558

[7] Pearce EJ, Sher A. Functional dichotomy in the CD4+T cell response to Schistosoma mansoni. Experimental Parasitology. 1991 7;73(1):110–6. 10.1016/0014-4894(91)90014-n 1711476

[8] Khairallah C, Chu TH, Sheridan BS. Tissue Adaptations of Memory and Tissue-Resident Gamma Delta T Cells. Front Immunol. 2018 11 27;9:2636 10.3389/fimmu.2018.02636 30538697 PMC6277633

[9] Nielsen MM, Witherden DA, Havran WL. γδ T cells in homeostasis and host defence of epithelial barrier tissues. Nat Rev Immunol. 2017 12;17(12):733–45. 10.1038/nri.2017.101 28920588 PMC5771804

[10] Božić F, Forčić D, Mažuran R, Marinculić A, Kozarić Z, Stojčević D. γδ TCR+ intestinal intraepithelial lymphocytes (i-IEL) in reaction against intestinal nematode. Comp Immun Microbiol Infect Dis. 1998;21:201–14. 10.1016/s0147-9571(98)00014-9 9681243

[11] Bozic F, Marinculic A, Durakovic E. Analysis of intestinal intraepithelial lymphocyte populations in experimental Trichinella spiralis infection of mice. FOLIA PARASIT. 2000 3 1;47(1):55–9. 10833017

[12] Inagaki-Ohara K, Sakamoto Y, Dohi T, Smith AL. γδ T cells play a protective role during infection with Nippostrongylus brasiliensis by promoting goblet cell function in the small intestine: Importance of γδ T cells in protection against intestinal nematode infection. Immunology. 2011 12;134(4):448–58. 10.1111/j.1365-2567.2011.03503.x 22044210 PMC3230798

[13] Hammerich L, Tacke F. Role of gamma-delta T cells in liver inflammation and fibrosis. WJGP. 2014;5(2):107 10.4291/wjgp.v5.i2.107 24891982 PMC4025070

[14] Chen D, Luo X, Xie H, Gao Z, Fang H, Huang J. Characteristics of IL-17 induction by Schistosoma japonicum infection in C57BL/6 mouse liver. Immunology. 2013 8;139(4):523–32. 10.1111/imm.12105 23551262 PMC3719069

[15] Yu X, Luo X, Xie H, Chen D, Li L, Wu F, et al Characteristics of γδ T cells in Schistosoma japonicum-infected mouse mesenteric lymph nodes. Parasitol Res. 2014 9;113(9):3393–401. 10.1007/s00436-014-4004-8 24994455

[16] Zheng L, Hu Y, Wang Y, Huang X, Xu Y, Shen Y, et al Recruitment of Neutrophils Mediated by Vγ2 γδ T Cells Deteriorates Liver Fibrosis Induced by Schistosoma japonicum Infection in C57BL/6 Mice. Adams JH, editor. Infect Immun. 2017 8;85(8):e01020–16, /iai/85/8/e01020-16.atom. 10.1128/IAI.01020-16 28507072 PMC5520426

[17] Chen D, Xie H, Luo X, Yu X, Fu X, Gu H, et al Roles of Th17 cells in pulmonary granulomas induced by Schistosoma japonicum in C57BL/6 mice. Cellular Immunology. 2013 9;285(1–2):149–57. 10.1016/j.cellimm.2013.09.008 24212062

[18] Sandor M, Sperling AI, Cook GA, Weinstock JV, Bluestone JA. Two waves of gamma delta T cells expressing different V delta genes are recruited into schistosome-induced liver granulomas. J Immunol. 1995;55:275–84. 7602105

[19] Schondelmaier S, Wesch D, Pechhold K, Kabelitz D. Vγ gene usage in peripheral blood γδ T cells. Immunology Letters. 1993;38. 10.1016/0165-2478(93)90176-3 8294139

[20] Vantourout P, Hayday A. Six-of-the-best: unique contributions of γδ T cells to immunology. Nat Rev Immunol. 2013 2;13(2):88–100. 10.1038/nri3384 23348415 PMC3951794

[21] Bukowski JF, Morita CT, Band H. Crucial Role of TCRγ Chain Junctional Region in Prenyl Pyrophosphate Antigen Recognition by γδ T Cells. J Immunol. 1998;161:286–93. 9647235

[22] Chen ZW, Letvin NL. Vγ2Vδ2+ T cells and anti-microbial immune responses. Microbes and Infection. 2003 5;5(6):491–8. 10.1016/s1286-4579(03)00074-1 12758278 PMC2873077

[23] Chen Z. Adaptive immune response of Vγ2Vδ2 T cells: a new paradigm. Trends in Immunology. 2003 4;24(4):213–9. 10.1016/s1471-4906(03)00032-2 12697454 PMC2869283

[24] Born WK, Harshan K. The role of y6 T lymphocytes in infection. Current Opinion in Immunology. 1991;3:455–9. 10.1016/0952-7915(91)90002-i 1836730

[25] Schwartz E, Rosenthal E, Bank I. Gamma delta T cells in non-immune patients during primary schistosomal infection: Gamma delta T cells in acute schistosomiasis. Immun Inflamm Dis. 2014 6;2(1):56–61. 10.1002/iid3.18 25400925 PMC4220667

[26] Morita CT, Lee HK, Leslie DS, Tanaka Y, Bukowski JF, Märker-Hermann E. Recognition of nonpeptide prenyl pyrophosphate antigens by human γd T cells. Microbes and Infection. 1999;12. 10594981

[27] Brooker S, Hotez PJ, Bundy DAP. The Global Atlas of Helminth Infection: Mapping the Way Forward in Neglected Tropical Disease Control. Aksoy S, editor. PLoS Negl Trop Dis. 2010 7 27;4(7):e779 10.1371/journal.pntd.0000779 20668545 PMC2910703

[28] Salgame P, Yap GS, Gause WC. Effect of helminth-induced immunity on infections with microbial pathogens. Nat Immunol. 2013 11;14(11):1118–26. 10.1038/ni.2736 24145791 PMC4955540

[29] Babu S, Nutman TB. Helminth-Tuberculosis Co-infection: An Immunologic Perspective. Trends in Immunology. 2016 9;37(9):597–607. 10.1016/j.it.2016.07.005 27501916 PMC5003706

[30] McLaughlin TA, Khayumbi J, Ongalo J, Tonui J, Campbell A, Allana S, et al CD4 T Cells in Mycobacterium tuberculosis and Schistosoma mansoni Co-infected Individuals Maintain Functional TH1 Responses. Front Immunol. 2020 2 7;11:127 10.3389/fimmu.2020.00127 32117277 PMC7020828

[31] Gaspard J, Usey MM, Fredericks-James M, Sanchez MJ, Atkins L, Campbell CH, et al Survey of Schistosomiasis in Saint Lucia: Evidence for Interruption of Transmission. The American Journal of Tropical Medicine and Hygiene [Internet]. 2020 2 10 [cited 2020 Feb 17]; Available from: http://www.ajtmh.org/content/journals/10.4269/ajtmh.19-0904 10.4269/ajtmh.19-0904 PMC7124901 32043449

[32] Lahm H-W, Stein S. Characterization of recombinant human interleukin-2 with micromethods. Journal of Chromatography A. 1985 6;326:357–61. 10.1016/s0021-9673(01)87461-6 3875623

[33] Finak G, McDavid A, Chattopadhyay P, Dominguez M, De Rosa S, Roederer M, et al Mixture models for single-cell assays with applications to vaccine studies. Biostatistics. 2014 1 1;15(1):87–101. 10.1093/biostatistics/kxt024 23887981 PMC3862207

[34] Lin L, Finak G, Ushey K, Seshadri C, Hawn TR, Frahm N, et al COMPASS identifies T-cell subsets correlated with clinical outcomes. Nat Biotechnol. 2015 6;33(6):610–6. 10.1038/nbt.3187 26006008 PMC4569006

[35] WHO | Schistosomiasis: progress report 2001–2011, strategic plan 2012–2020 [Internet]. WHO. [cited 2019 Jun 14]. Available from: http://www.who.int/schistosomiasis/resources/9789241503174/en/

[36] Caldas IR, Campi-Azevedo AC, Oliveira LFA, Silveira AMS, Oliveira RC, Gazzinelli G. Human schistosomiasis mansoni: Immune responses during acute and chronic phases of the infection. Acta Tropica. 2008 11;108(2–3):109–17. 10.1016/j.actatropica.2008.05.027 18577364

[37] Lambertucci JR, Parra JC, Colley DG, Garcia AA, Gazzinelli G, Katz N, et al Immune Responses During Human Schistosomiasis: XII. Differential Responsiveness in Patients with Hepatosplenic Disease. The American Journal of Tropical Medicine and Hygiene. 1986 7 1;35(4):793–802. 3089040

[38] Naus CWA, Kimani G, Ouma JH, Fulford AJC, Webster M, van Dam GJ, et al Development of Antibody Isotype Responses to Schistosoma mansoni in an Immunologically Naive Immigrant Population: Influence of Infection Duration, Infection Intensity, and Host Age. Kaufmann SHE, editor. Infection and Immunity. 1999;67(7):3444–51. 10.1128/IAI.67.7.3444-3451.1999 10377125 PMC116530

[39] Almeida CA, Leite MF, Goes AM. Signal transduction events in human peripheral blood mononuclear cells stimulated by schistosoma mansoni antigens. Human Immunology. 2001 10;62(10):1159–66. 10.1016/s0198-8859(01)00302-0 11600225

[40] Oliveira-Prado R, Caldas IR, Teixeira-Carvalho A, Andrade MV, Fares RCG, Portugal LM, et al Cytokine profile, proliferation and phosphorylation of ERK1/2 and Akt in circulating mononuclear cells from individuals during the chronic intestinal phase of Schistosomiasis mansoni infection. BMC Infect Dis. 2012 12;12(1):380. 23270458 10.1186/1471-2334-12-380 PMC3549743

[41] Joseph S, Jones FM, Kimani G, Mwatha JK, Kamau T, Kazibwe F, et al Cytokine Production in Whole Blood Cultures from a Fishing Community in an Area of High Endemicity for Schistosoma mansoni in Uganda: the Differential Effect of Parasite Worm and Egg Antigens. IAI. 2004 2;72(2):728–34. 10.1128/IAI.72.2.728-734.2004 PMC321598 14742514

[42] Marguerite M, Gallissot M-C, Diagne M, Moreau C, Diakkhate M-M, Roberts M, et al Cellular immune responses of a Senegalese community recently exposed to Schistosoma mansoni: correlations of infection level with age and inflammatory cytokine production by soluble egg antigen-specific cells. Trop Med Int Health. 1999 8;4(8):530–43. 10.1046/j.1365-3156.1999.00443.x 10499076

[43] Ondigo BN, Ndombi EM, Nicholson SC, Oguso JK, Carter JM, Kittur N, et al Functional Studies of T Regulatory Lymphocytes in Human Schistosomiasis in Western Kenya. The American Journal of Tropical Medicine and Hygiene. 2018 6 6;98(6):1770–81. 10.4269/ajtmh.17-0966 29692308 PMC6086154

[44] Chen ZW. Immune regulation of γδ T cell responses in mycobacterial infections. Clinical Immunology. 2005 9;116(3):202–7. 10.1016/j.clim.2005.04.005 16087145 PMC2869281

[45] Sakari SSW, Mbugua AK, Mkoji GM. Prevalence of Soil-Transmitted Helminthiases and Schistosomiasis in Preschool Age Children in Mwea Division, Kirinyaga South District, Kirinyaga County, and Their Potential Effect on Physical Growth. Journal of Tropical Medicine. 2017;2017:1–12. 10.1155/2017/1013802 PMC5613645 29138640

[46] Odiere MR, Rawago FO, Ombok M, Secor WE, Karanja DM, Mwinzi PN, et al High prevalence of schistosomiasis in Mbita and its adjacent islands of Lake Victoria, western Kenya. Parasites Vectors. 2012 12;5(1):278. 23206383 10.1186/1756-3305-5-278 PMC3523971

[47] Salgame P, Geadas C, Collins L, Jones-López E, Ellner JJ. Latent tuberculosis infection–Revisiting and revising concepts. Tuberculosis. 2015 7;95(4):373–84. 10.1016/j.tube.2015.04.003 26038289

[48] Pai M, Riley LW, Jr JMC. Interferon-γ assays in the immunodiagnosis of tuberculosis: a systematic review. The Lancet Infectious Diseases. 2004;4:761–76. 10.1016/S1473-3099(04)01206-X 15567126

[49] Abubakar I, Drobniewski F, Southern J, Sitch AJ, Jackson C, Lipman M, et al Prognostic value of interferon-γ release assays and tuberculin skin test in predicting the development of active tuberculosis (UK PREDICT TB): a prospective cohort study. The Lancet Infectious Diseases. 2018 10;18(10):1077–87. 10.1016/S1473-3099(18)30355-4 30174209 PMC6192014

[50] Bendelac A, Savage PB, Teyton L. The Biology of NKT Cells. Annu Rev Immunol. 2007 4;25(1):297–336. 17150027 10.1146/annurev.immunol.25.022106.141711

[51] Cowley SC. MAIT cells and pathogen defense. Cell Mol Life Sci. 2014 12;71(24):4831–40. 10.1007/s00018-014-1708-y 25164578 PMC11113923

[52] Kjer-Nielsen L, Corbett AJ, Chen Z, Liu L, Mak JY, Godfrey DI, et al An overview on the identification of MAIT cell antigens. Immunol Cell Biol. 2018 7;96(6):573–87. 10.1111/imcb.12057 29656544

[53] Xiao X, Cai J. Mucosal-Associated Invariant T Cells: New Insights into Antigen Recognition and Activation. Front Immunol. 2017 11 10;8:1540 10.3389/fimmu.2017.01540 29176983 PMC5686390

[54] Carding SR, Egan PJ. γδ T cells: functional plasticity and heterogeneity. Nat Rev Immunol. 2002 5;2(5):336–45. 10.1038/nri797 12033739

[55] Kalyan S, Kabelitz D. Defining the nature of human γδ T cells: a biographical sketch of the highly empathetic. Cell Mol Immunol. 2013 1;10(1):21–9. 10.1038/cmi.2012.44 23085947 PMC4003173

[56] Adams EJ, Gu S, Luoma AM. Human gamma delta T cells: Evolution and ligand recognition. Cellular Immunology. 2015 7;296(1):31–40. 10.1016/j.cellimm.2015.04.008 25991474 PMC4466157

[57] Lawand M, Déchanet-Merville J, Dieu-Nosjean M-C. Key Features of Gamma-Delta T-Cell Subsets in Human Diseases and Their Immunotherapeutic Implications. Front Immunol. 2017 6 30;8:761 10.3389/fimmu.2017.00761 28713381 PMC5491929

[58] Lalor SJ, McLoughlin RM. Memory γδ T Cells–Newly Appreciated Protagonists in Infection and Immunity. Trends in Immunology. 2016 10;37(10):690–702. 10.1016/j.it.2016.07.006 27567182

[59] Shen L, Frencher J, Huang D, Wang W, Yang E, Chen CY, et al Immunization of Vγ2Vδ2 T cells programs sustained effector memory responses that control tuberculosis in nonhuman primates. Proc Natl Acad Sci USA. 2019 3 26;116(13):6371–8. 10.1073/pnas.1811380116 30850538 PMC6442559

[60] Bahia-Oliveira LMG, Gazzinelli G, Eloi-Santos SM, Cunha-Melo JR, Alves-Oliveira LF, Silveira AMS, et al Differential cellular reactivity to adult worm antigens of patients with different clinical forms of schistosomiasis mansoni. Transactions of the Royal Society of Tropical Medicine and Hygiene. 1992 1;86(1):57–61. 10.1016/0035-9203(92)90441-e 1566307

[61] Rajkovic A, Simonsen JN, Davis RE, Rottman FM. Molecular cloning and sequence analysis of 3-hydroxy-3-methylglutaryl-coenzyme A reductase from the human parasite Schistosoma mansoni. Proceedings of the National Academy of Sciences. 1989 11 1;86(21):8217–21. 10.1073/pnas.86.21.8217 PMC298251 2813388

[62] Venancio TM, DeMarco R, Almeida GT, Oliveira KC, Setubal JC, Verjovski-Almeida S. Analysis of Schistosoma mansoni genes shared with Deuterostomia and with possible roles in host interactions. BMC Genomics. 2007;8(1):407. 17996068 10.1186/1471-2164-8-407 PMC2194728

[63] Ziniel PD, Desai J, Cass CL, Gatto C, Oldfield E, Williams DL. Characterization of Potential Drug Targets Farnesyl Diphosphate Synthase and Geranylgeranyl Diphosphate Synthase in Schistosoma mansoni. Antimicrob Agents Chemother. 2013 12;57(12):5969–76. 10.1128/AAC.00699-13 24041901 PMC3837879

[64] Jankovic D, Wynn TA, Kullberg MC, Hieny S, Caspar P, James S, et al Optimal Vaccination Against Schistosoma mansoni Requires the Induction of Both B Cell- and IFN-γ-Dependent Effector Mechanisms. J Immunol. 1999 1 1;162(1):345 9886405

[65] Vimal IRC, Sher A, Carvalho OS, Massara CL, Eloi-Santos’ SM, Pearce EJ, et al Interferon-y production by peripheral blood mononuclear cells from residents of an area endemic for Schistosoma mansoni. Transactions of the Royal Society of Tropical Medicine and Hygiene. 1994;88:466–70. 10.1016/0035-9203(94)90436-7 7570847

[66] Pearce EJ, Sher A. Mechanisms of immune evasion in schistosomiasis. Contributions to microbiology and immunology. 1987;8:219–32. 3304833

[67] Molehin AJ, Rojo JU, Siddiqui SZ, Gray SA, Carter D, Siddiqui AA. Development of a schistosomiasis vaccine. Expert Review of Vaccines. 2016 5 3;15(5):619–27. 10.1586/14760584.2016.1131127 26651503 PMC5070536

[68] Corrêa-Oliveira R, Rodrigues Caldas I, Gazzinelli G. Natural versus Drug-induced Resistance in Schistosoma mansoni Infection. Parasitology Today. 2000 9;16(9):397–9. 10.1016/s0169-4758(00)01740-3 10951600

