# Supplementary material for: Adults from Kisumu, Kenya have robust γδ T cell responses to Schistosoma mansoni, which are modulated by tuberculosis
Source: PLoS Negl Trop Dis. 2020 Oct 12;14(10):e0008764. doi: 10.1371/journal.pntd.0008764 (PMC7580987; doi:10.1371/journal.pntd.0008764)
Supplement: S1 Fig — (A) In this sample gating for the overnight ICS assay, cells were first gated for singlets (FSC-H vs. FSC- A) and lymphocytes (SSC-A vs. FSC-A). The lymphocyte gate is further analyzed for their uptake of the Zombie IR Live/Dead stain to determine live versus dead cells and their expression of CD3 (Zombie Near-IRlo, CD3+). CD4 and CD8 surface expression is then determined from this gated population. (B) In this sample gating for the Proliferation ICS assay, cells were first gated for singlets (FSC-H vs. FSC- A) and lymphocytes (SSC-A vs. FSC-A). The lymphocyte gate is further analyzed for their uptake of the Zombie IR Live/Dead stain to determine live versus dead cells (Zombie Near-IRlo). Live cells are then gated for their expression of CD3 (CD3+). CD4, CD8, and γδ surface expression is then determined from this gated population. CD4 T cells were defined as CD3+CD4+CD8- lymphocytes, CD8 T cells were defined as CD3+CD4-CD8+ lymphocytes, and a third population of T cells were defined as CD3+CD4-CD8- lymphocytes. (PDF) [file pntd.0008764.s001.pdf]

Supporting Information

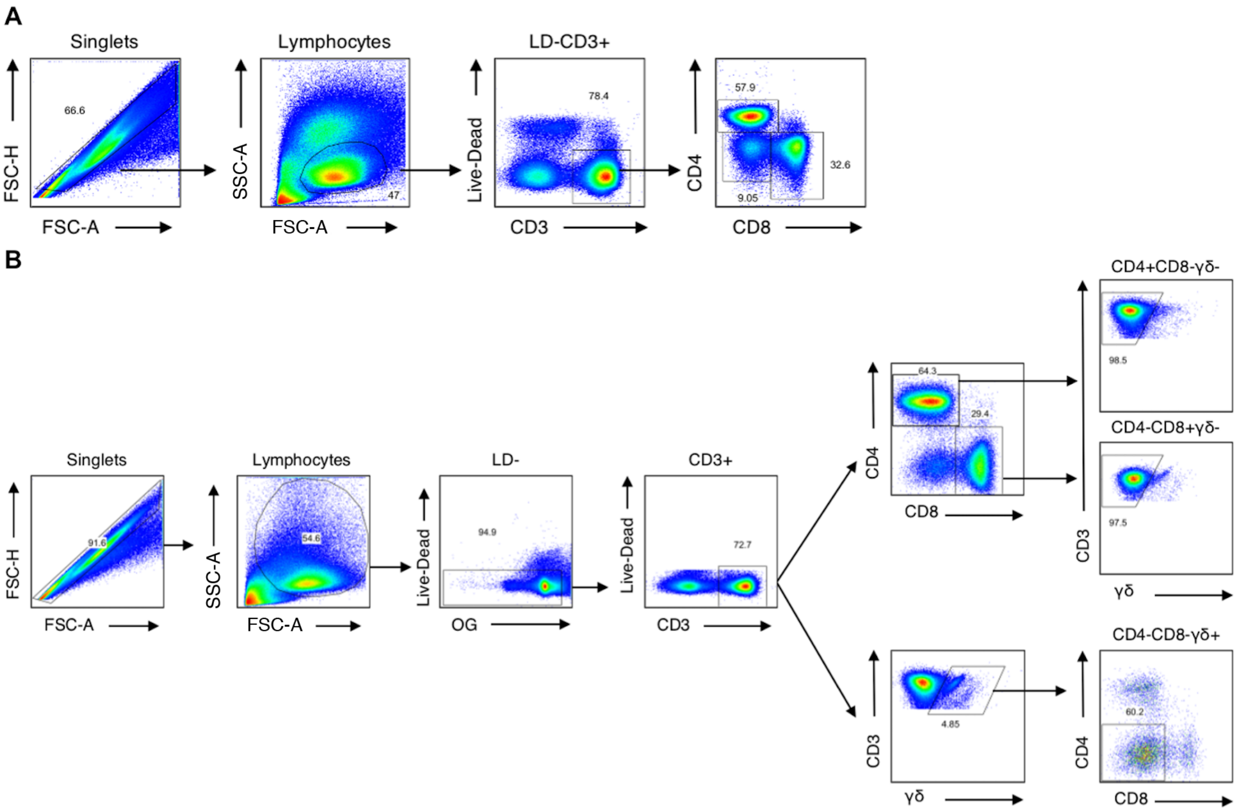

S1 Fig. Gating strategy for flow cytometry analysis. (A) In this sample gating for the overnight ICS assay, cells were first gated for singlets (FSC-H vs. FSC-A) and lymphocytes (SSC-A vs. FSC-A). The lymphocyte gate is further analyzed for their uptake of the Zombie IR Live/Dead stain to determine live versus dead cells and their expression of CD3 (Zombie Near-IR^{lo}, CD3+). CD4 and CD8 surface expression is then determined from this gated population. **(B)** In this sample gating for the Proliferation ICS assay, cells were first gated for singlets (FSC-H vs. FSC- A) and lymphocytes (SSC-A vs. FSC-A). The lymphocyte gate is further analyzed for their uptake of the Zombie IR Live/Dead stain to determine live versus dead cells (Zombie Near-IR^{lo}). Live cells are then gated for their expression of CD3 (CD3+). CD4, CD8, and γδ surface expression is then determined from this gated population. CD4 T cells were defined as CD3+CD4+CD8− lymphocytes, CD8 T cells were defined as CD3+CD4−CD8+ lymphocytes, and a third population of T cells were defined as CD3+CD4−CD8− lymphocytes.
