# Supplementary material for: Adults from Kisumu, Kenya have robust γδ T cell responses to Schistosoma mansoni, which are modulated by tuberculosis
Source: PLoS Negl Trop Dis. 2020 Oct 12;14(10):e0008764. doi: 10.1371/journal.pntd.0008764 (PMC7580987; doi:10.1371/journal.pntd.0008764)
Supplement: S2 Fig — PBMC from individuals in each group were stimulated and analyzed by flow cytometry as described in Fig 1. Intracellular expression of IFNγ, TNFα, IL-4, and IL-13 was measured by flow cytometry. (A) Frequency of total cytokine+ cells within each designated cell type are reported. (B) Frequency of each combination of cytokine+ cells using a Boolean gating strategy within each cell type are reported. Boxes represent the median and interquartile ranges; whiskers represent the 1.5*IQR. Differences in the cytokine frequency between SEA and SWAP were assessed using a Mann-Whitney U test. **** p<0.0001; *** p<0.001; ** p< 0.01; * p< 0.05. (PDF) [file pntd.0008764.s002.pdf]

Supporting Information

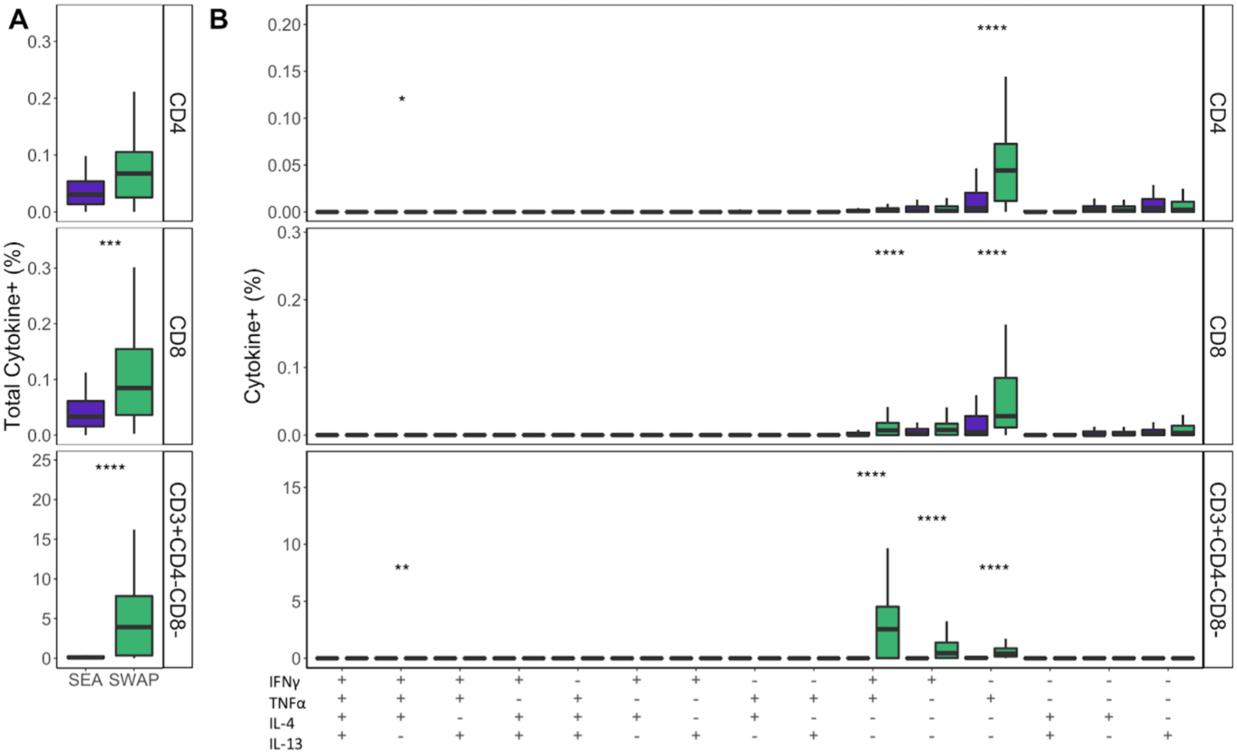

S2 Fig. Cytokine frequencies are higher following SWAP stimulation than SEA stimulation. PBMC from individuals in each group were stimulated and analyzed by flow cytometry as described in Fig 1. Intracellular expression of IFNγ, TNFα, IL-4, and IL-13 was measured by flow cytometry. **(A)** Frequency of total cytokine+ cells within each designated cell type are reported. **(B)** Frequency of each combination of cytokine+ cells using a Boolean gating strategy within each cell type are reported. Boxes represent the median and interquartile ranges; whiskers represent the 1.5*IQR. Differences in the cytokine frequency between SEA and SWAP were assessed using a Mann-Whitney U test. **** $p<0.0001$ *** $p<0.001$; ** $p< 0.01$; * $p< 0.05$
