# Supplementary material for: Adults from Kisumu, Kenya have robust γδ T cell responses to Schistosoma mansoni, which are modulated by tuberculosis
Source: PLoS Negl Trop Dis. 2020 Oct 12;14(10):e0008764. doi: 10.1371/journal.pntd.0008764 (PMC7580987; doi:10.1371/journal.pntd.0008764)
Supplement: S3 Fig — PBMC from individuals in each group were stimulated with PMA and analyzed by flow cytometry as described in Fig 1. Intracellular expression of IFNγ, TNFα, IL-4, and IL-13 was measured by flow cytometry. (A) ICS data were analyzed using COMPASS and the results from each cytokine subset are displayed as a heatmap. Rows represent study subjects and columns represent cytokine combinations. The intensity of shading represents the probability of detecting a response above background. (C) Subject-specific COMPASS results were summarized for 63 individuals using the functionality and polyfunctionality scores. Scores from CD4, CD8 and CD3+CD4-CD8- T cell subsets were aggregated across all groups. Boxes represent the median and interquartile ranges; whiskers represent the 1.5*IQR. Differences between the scores of each T cell subset were assessed using a Kruskal-Wallis test with Nemenyi correction for multiple pairwise comparisons. *** p<0.001; ** p< 0.01; * p< 0.05. (PDF) [file pntd.0008764.s003.pdf]

Supporting Information

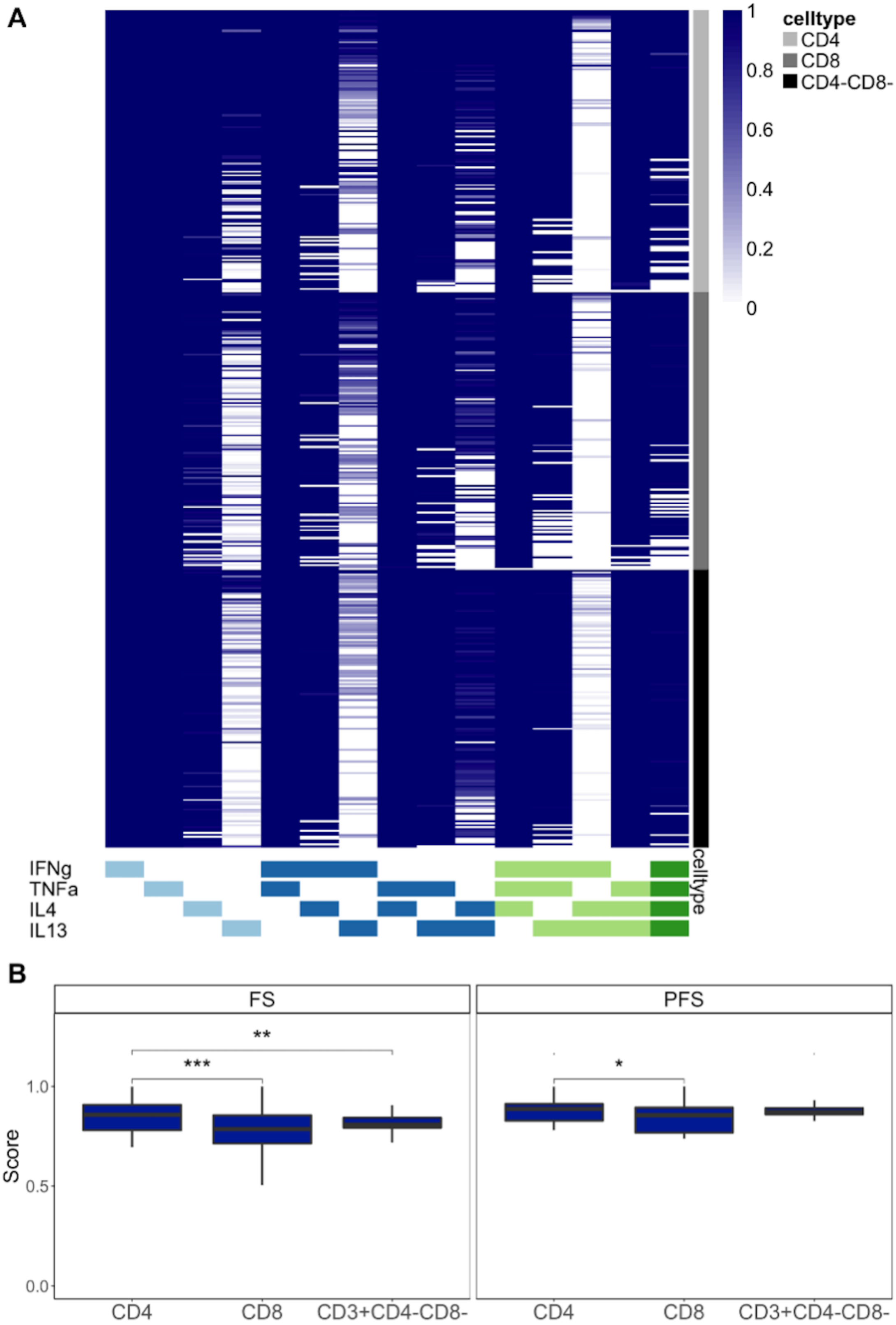

S3 Fig. CD4 T cells have a greater functional response to PMA/ionomycin than CD8 and CD3+CD4−CD8− T cells. PBMC from individuals in each group were stimulated with PMA and analyzed by flow cytometry as described in Fig 1. Intracellular expression of IFNγ, TNFα, IL-4, and IL-13 was measured by flow cytometry. (**A**) ICS data were analyzed using COMPASS and the results from each cytokine subset are displayed as a heatmap. Rows represent study subjects and columns represent cytokine combinations. The intensity of shading represents the probability of detecting a response above background. (**C**) Subject-specific COMPASS results were summarized for 63 individuals using the functionality and polyfunctionality scores. Scores from CD4, CD8 and CD3+CD4−CD8− T cell subsets were aggregated across all groups. Boxes represent the median and interquartile ranges; whiskers represent the 1.5*IQR. Differences between the scores of each T cell subset were assessed using a Kruskal-Wallis test with Nemenyi correction for multiple pairwise comparisons. *** $p<0.001$; ** $p< 0.01$; * $p< 0.05$
