# Supplementary material for: Adults from Kisumu, Kenya have robust γδ T cell responses to Schistosoma mansoni, which are modulated by tuberculosis
Source: PLoS Negl Trop Dis. 2020 Oct 12;14(10):e0008764. doi: 10.1371/journal.pntd.0008764 (PMC7580987; doi:10.1371/journal.pntd.0008764)
Supplement: S4 Fig — PBMC from individuals in each group were stimulated with PMA and analyzed by flow cytometry as described in Fig 1. Intracellular expression of IFNγ, TNFα, IL-4, and IL-13 was measured by flow cytometry. Frequency of each combination of cytokine+ cells using a Boolean gating strategy within each cell type are reported. Boxes represent the median and interquartile ranges; whiskers represent the 1.5*IQR. (PDF) [file pntd.0008764.s004.pdf]

Supporting Information

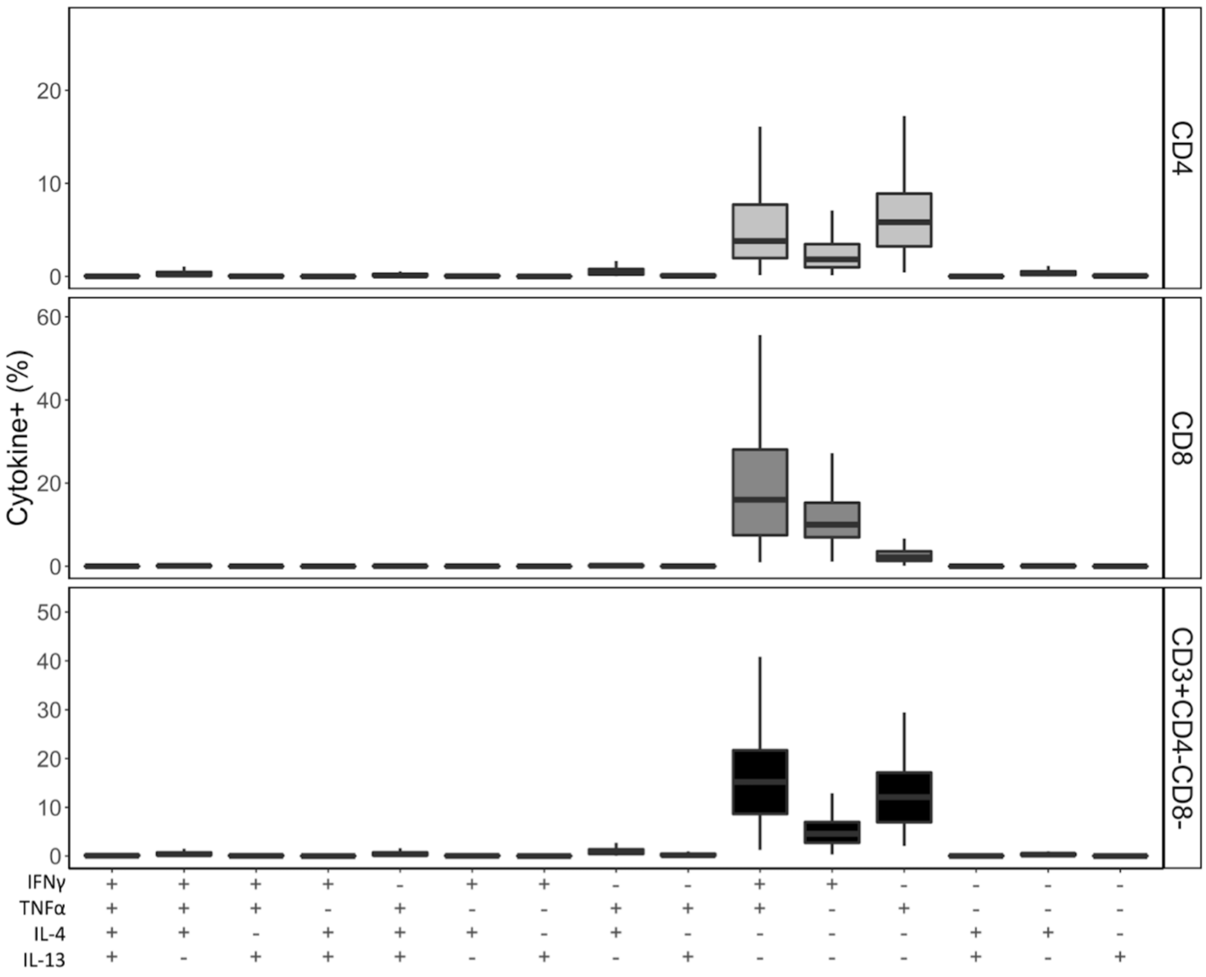

S4 Fig. IFNγ and TNFα are produced in response to PMA and Ionomycin across T cell types. PBMC from individuals in each group were stimulated with PMA and analyzed by flow cytometry as described in Fig 1. Intracellular expression of IFNγ, TNFα, IL-4, and IL-13 was measured by flow cytometry. Frequency of each combination of cytokine+ cells using a Boolean gating strategy within each cell type are reported. Boxes represent the median and interquartile ranges; whiskers represent 1.5*IQR.
