# Supplementary material for: Adults from Kisumu, Kenya have robust γδ T cell responses to Schistosoma mansoni, which are modulated by tuberculosis
Source: PLoS Negl Trop Dis. 2020 Oct 12;14(10):e0008764. doi: 10.1371/journal.pntd.0008764 (PMC7580987; doi:10.1371/journal.pntd.0008764)
Supplement: S5 Fig — PBMC from individuals in each of four groups defined by Mtb and SM infection status (N, n = 12; IGRA-, n = 12; IGRA+, n = 23; TB, n = 15) were incubated for 18 h in media alone (negative control) or stimulated with SEA or SWAP. Intracellular expression of IFNγ, TNFα, IL-4, and IL-13 was measured by flow cytometry and data were analyzed using COMPASS. (A-C) Functionality and polyfunctionality scores for CD4 (A), CD8 (B), and CD3+CD4-CD8- (C) T cells. Boxes represent the median and interquartile ranges; whiskers represent the 1.5*IQR. Differences in the scores of each T cell subset were assessed using a Kruskal-Wallis test with Nemenyi correction for multiple pairwise comparisons. **** p<0.0001; *** p<0.001; ** p< 0.01; * p< 0.05. (PDF) [file pntd.0008764.s005.pdf]

Supporting Information

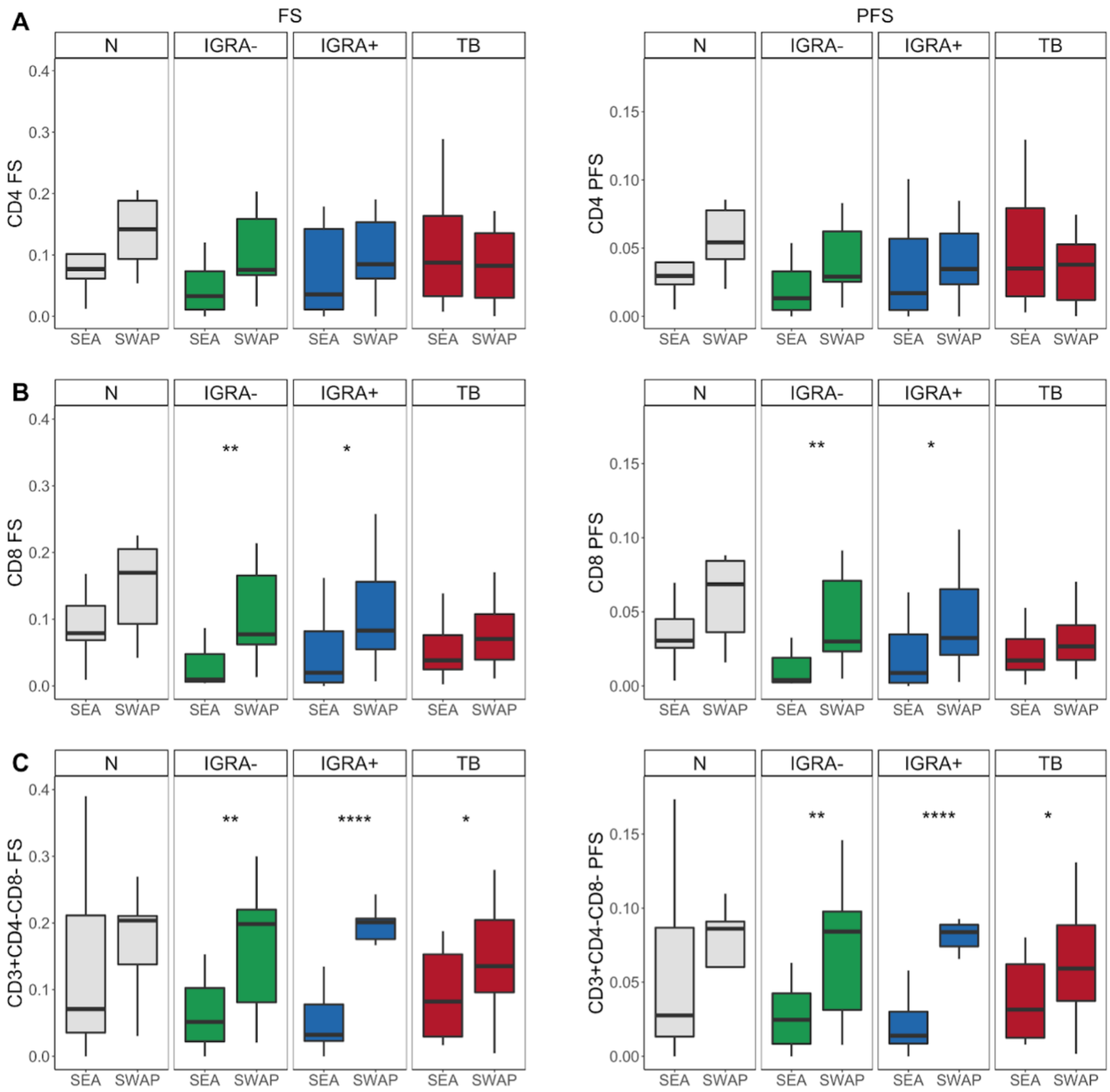

S5 Fig. SWAP functionality and polyfunctionality scores are higher than SEA responses in IGRA- and IGRA+ groups. PBMC from individuals in each of four groups defined by Mtb and SM infection status (N, n=12; IGRA-, n=12; IGRA+, n=23; TB, n=15) were incubated for 18 h in media alone (negative control) or stimulated with SEA or SWAP. Intracellular expression of IFNγ, TNFα, IL-4, and IL-13 was measured by flow cytometry and data were analyzed using COMPASS. **(A-C)** Functionality and polyfunctionality scores for CD4 **(A)**, CD8 **(B)**, and CD3+CD4−CD8− **(C)** T cells. Boxes represent the median and interquartile ranges; whiskers represent the 1.5*IQR. Differences in the scores of each T cell subset were assessed using a Kruskal-Wallis test with Nemenyi correction for multiple pairwise comparisons. **** $p<0.0001$; *** $p<0.001$; ** $p< 0.01$; * $p< 0.05$
