# Supplementary material for: Adults from Kisumu, Kenya have robust γδ T cell responses to Schistosoma mansoni, which are modulated by tuberculosis
Source: PLoS Negl Trop Dis. 2020 Oct 12;14(10):e0008764. doi: 10.1371/journal.pntd.0008764 (PMC7580987; doi:10.1371/journal.pntd.0008764)
Supplement: S6 Fig — PBMC samples obtained from individuals in each of four groups defined by Mtb and SMinfection status (N, n = 12; IGRA-, n = 12; IGRA+, n = 23; TB, n = 15) were incubated for 18 hours in media alone (negative control) or stimulated with SEA or SWAP. Intracellular expression of IFNγ, TNFα, IL-4, and IL-13 was measured by flow cytometry. COMPASS functionality scores among each T cell subset are reported. Boxes represent the median and interquartile ranges; whiskers represent the 1.5*IQR. Differences in the scores of each T cell subset were assessed using a Kruskal-Wallis test with Nemenyi correction for multiple pairwise comparisons. *** p<0.001; ** p< 0.01. (PDF) [file pntd.0008764.s006.pdf]

Supporting Information

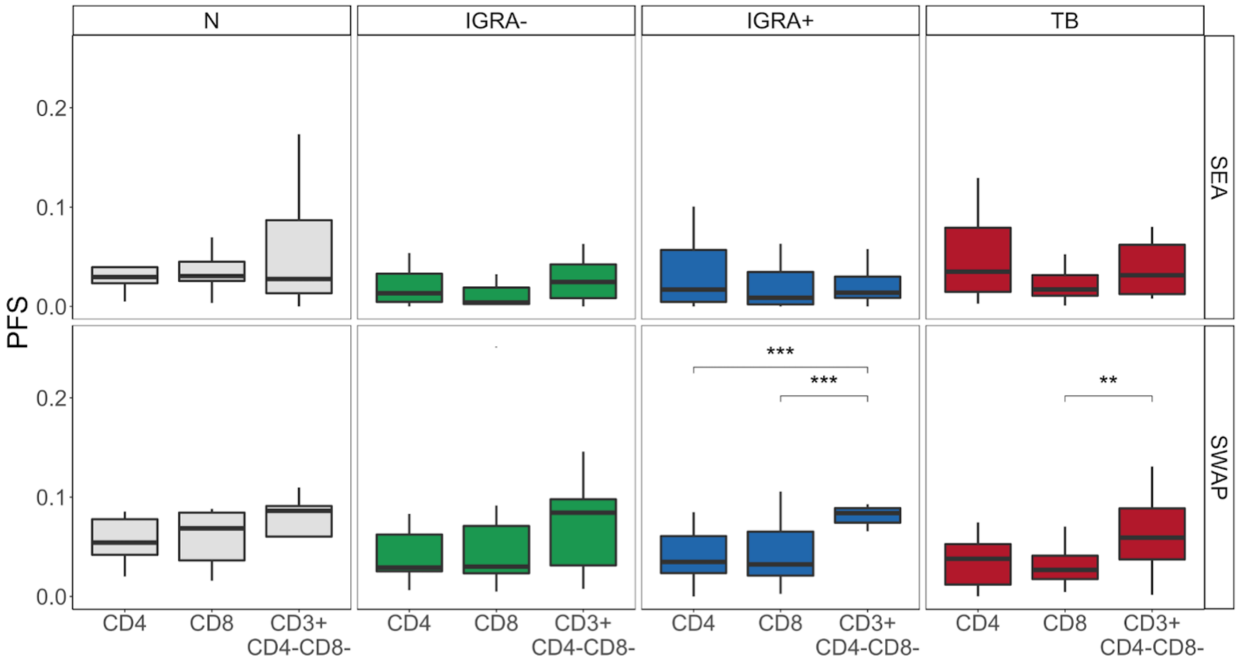

S6 Fig. CD3+CD4−CD8− T cells have a greater polyfunctional response to SWAP than CD4 and CD8 T cells in IGRA+ and TB individuals. PBMC samples obtained from individuals in each of four groups defined by Mtb and SM infection status (N, n=12; IGRA-, n=12; IGRA+, n=23; TB, n=15) were incubated for 18 hours in media alone (negative control) or stimulated with SEA or SWAP. Intracellular expression of IFNγ, TNFα, IL-4, and IL-13 was measured by flow cytometry. COMPASS functionality scores among each T cell subset are reported. Boxes represent the median and interquartile ranges; whiskers represent the 1.5*IQR. Differences in the scores of each T cell subset were assessed using a Kruskal-Wallis test with Nemenyi correction for multiple pairwise comparisons. *** $p<0.001$, ** $p< 0.01$
