# Supplementary material for: Adults from Kisumu, Kenya have robust γδ T cell responses to Schistosoma mansoni, which are modulated by tuberculosis
Source: PLoS Negl Trop Dis. 2020 Oct 12;14(10):e0008764. doi: 10.1371/journal.pntd.0008764 (PMC7580987; doi:10.1371/journal.pntd.0008764)
Supplement: S7 Fig — PBMC samples obtained from individuals in each of four groups defined by Mtb and SM infection status (N, n = 12; IGRA-, n = 12; IGRA+, n = 23; TB, n = 15) were incubated for 18 hours in media alone (negative control) or stimulated with SEA or SWAP. Intracellular expression of IFNγ, TNFα, IL-4, and IL-13 was measured by flow cytometry and data were analyzed using COMPASS. (A-C) SEA-specific FS and PFS for CD4 (A), CD8 (B), and CD4-CD8- (C) T cells. (D-F) SWAP-specific FS and PFS for CD4 (D), CD8 (E), and CD4-CD8- (F) T cells. Boxes represent the median and interquartile ranges; whiskers represent the 1.5*IQR. Differences in the scores of each T cell subset were assessed using a Kruskal-Wallis test with Nemenyi correction for multiple pairwise comparisons. ** p< 0.01. (PDF) [file pntd.0008764.s007.pdf]

Supporting Information

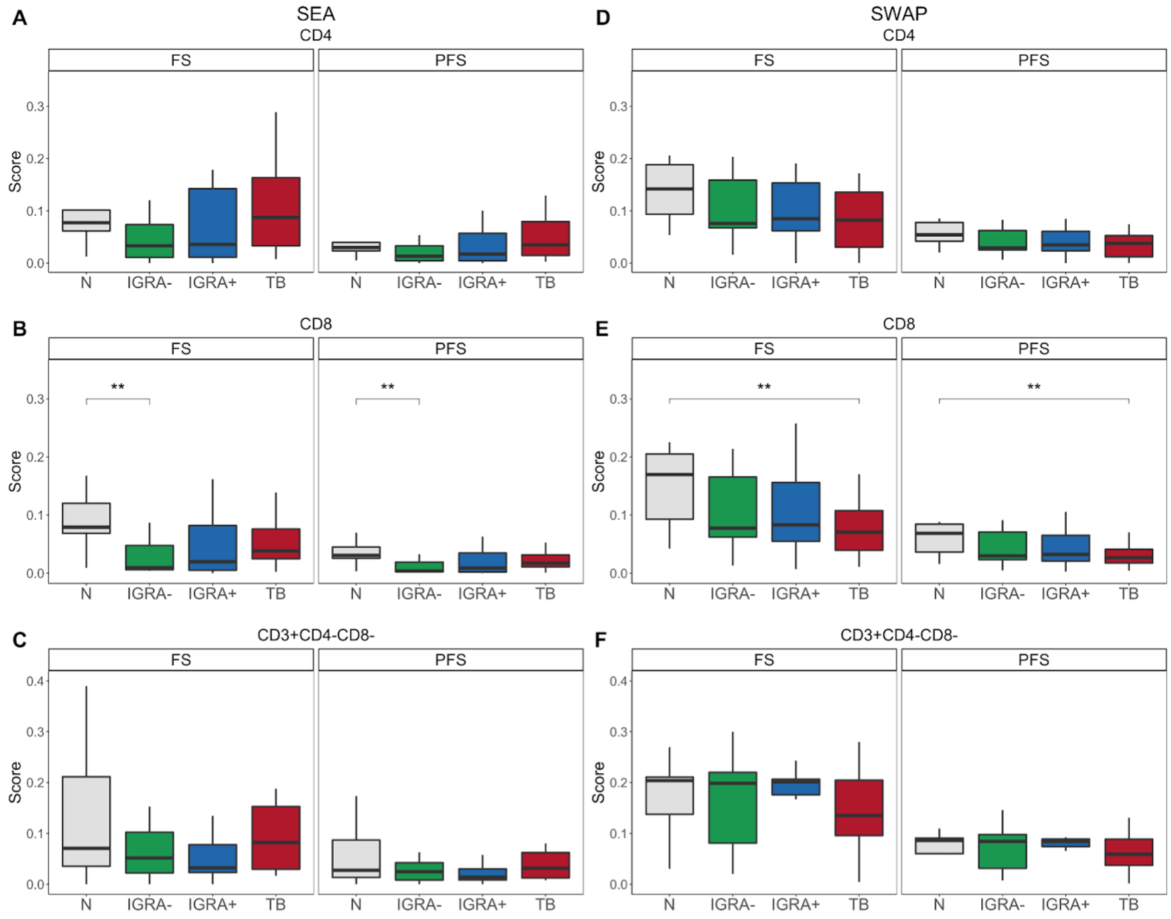

S7 Fig. CD8 T cell responses to both SEA and SWAP are higher in Naïve individuals. PBMC samples obtained from individuals in each of four groups defined by Mtb and SM infection status (N, n=12; IGRA-, n=12; IGRA+, n=23; TB, n=15) were incubated for 18 hours in media alone (negative control) or stimulated with SEA or SWAP. Intracellular expression of IFNγ, TNFα, IL-4, and IL-13 was measured by flow cytometry and data were analyzed using COMPASS. **(A-C)** SEA-specific FS and PFS for CD4 **(A)**, CD8 **(B)**, and CD4−CD8− **(C)** T cells. **(D-F)** SWAP-specific FS and PFS for CD4 **(D)**, CD8 **(E)**, and CD4−CD8− **(F)** T cells. Boxes represent the median and interquartile ranges; whiskers represent 1.5*IQR. Differences in the scores of each T cell subset were assessed using a Kruskal-Wallis test with Nemenyi correction for multiple pairwise comparisons. ** $p < 0.01$
