# Supplementary material for: Adults from Kisumu, Kenya have robust γδ T cell responses to Schistosoma mansoni, which are modulated by tuberculosis
Source: PLoS Negl Trop Dis. 2020 Oct 12;14(10):e0008764. doi: 10.1371/journal.pntd.0008764 (PMC7580987; doi:10.1371/journal.pntd.0008764)
Supplement: S8 Fig — Proliferation assays were performed as described in Fig 3. (A) Frequency of total CD4 T cells in the unstimulated (UN) condition (N, n = 10; IGRA-, n = 13; IGRA+, n = 24; TB, n = 16). (B-D) Frequency of proliferating (OGlo) CD4 T cells to SEB (B), SEA (C) and SWAP (D). Proliferation data are shown after subtraction of background proliferation in the UN condition. Boxes represent the median and interquartile ranges; whiskers represent the 1.5*IQR. Differences in the frequency of each proliferating CD4 T cell population between groups were assessed using a Kruskal-Wallis test with Nemenyi correction for multiple pairwise comparisons. (PDF) [file pntd.0008764.s008.pdf]

Supporting Information

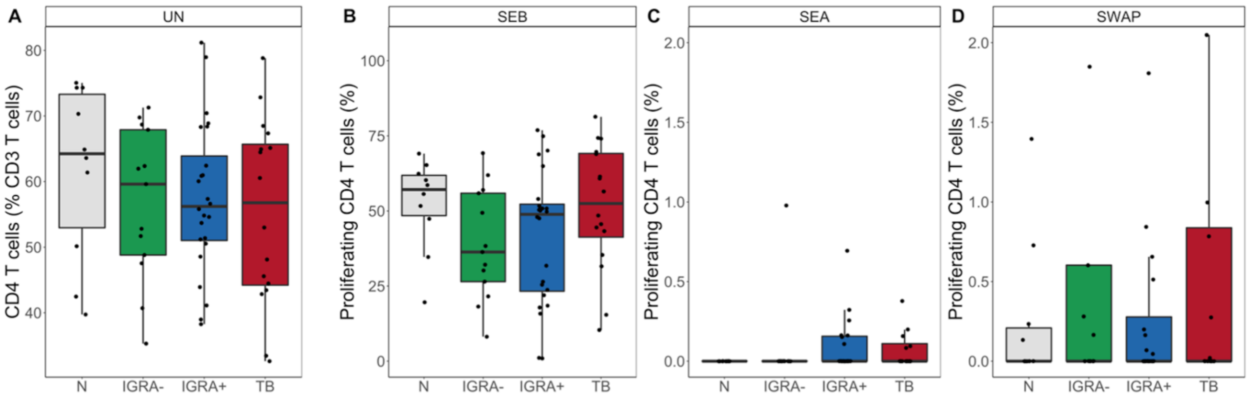

S8 Fig. SWAP-reactive CD4 T cell proliferation capacity is equivalent between groups. Proliferation assays were performed as described in Fig 3. **(A)** Frequency of total CD4 T cells in the unstimulated (UN) condition (N, n=10; IGRA-, n=13; IGRA+, n=24; TB, n=16). **(B-D)** Frequency of proliferating (OG^{lo}) CD4 T cells to SEB **(B),** SEA **(C)** and SWAP **(D)**. Proliferation data are shown after subtraction of background proliferation in the UN condition. Boxes represent the median and interquartile ranges; whiskers represent 1.5*IQR. Differences in the frequency of each proliferating CD4 T cell population between groups were assessed using a Kruskal-Wallis test with Nemenyi correction for multiple pairwise comparisons.
