# Supplementary material for: Adults from Kisumu, Kenya have robust γδ T cell responses to Schistosoma mansoni, which are modulated by tuberculosis
Source: PLoS Negl Trop Dis. 2020 Oct 12;14(10):e0008764. doi: 10.1371/journal.pntd.0008764 (PMC7580987; doi:10.1371/journal.pntd.0008764)
Supplement: S9 Fig — Proliferation assays were performed using PBMC obtained from individuals in each of four groups defined by Mtb and SM infection status (N, n = 10; IGRA-, n = 13; IGRA+, n = 24; TB, n = 16). Cells were labeled with Oregon Green (OG) and incubated for 5 days under the following conditions: media alone (negative control), SEB (positive control), SEA or SWAP. (A-C) Frequency of OGlo (proliferating) CD3+ T cells to SEB (A), SEA (B) and SWAP (C). Data are shown after subtraction of background proliferation in the unstimulated negative control condition. Boxes represent the median and interquartile ranges; whiskers represent the 1.5*IQR. Differences in the frequency of each proliferating CD3+ T cell population between groups were assessed using a Kruskal-Wallis test with Nemenyi correction for multiple pairwise comparisons. (PDF) [file pntd.0008764.s009.pdf]

Supporting Information

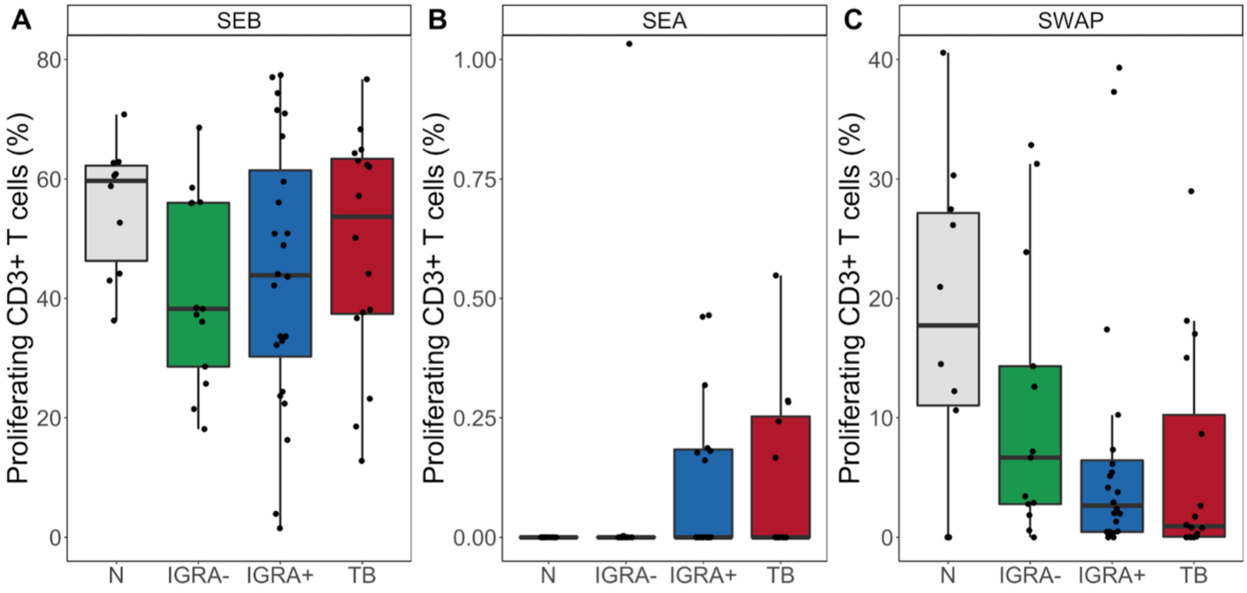

S9 Fig. Proliferation of total CD3+ T cells does not differ by Mtb and SM infection status. Proliferation assays were performed using PBMC obtained from individuals in each of four groups defined by Mtb and SM infection status (N, n=10; IGRA-, n=13; IGRA+, n=24; TB, n=16). Cells were labeled with Oregon Green (OG) and incubated for 5 days under the following conditions: media alone (negative control), SEB (positive control), SEA or SWAP. **(A-C)** Frequency of OG^{lo} (proliferating) CD3+ T cells to SEB **(A)**, SEA **(B)** and SWAP **(C)**. Data are shown after subtraction of background proliferation in the unstimulated negative control condition. Boxes represent the median and interquartile ranges; whiskers represent 1.5*IQR. Differences in the frequency of each proliferating CD3+ T cell population between groups were assessed using a Kruskal-Wallis test with Nemenyi correction for multiple pairwise comparisons.
